# Scoping Review of the Zika Virus Literature

**DOI:** 10.1371/journal.pone.0156376

**Published:** 2016-05-31

**Authors:** Lisa A. Waddell, Judy D. Greig

**Affiliations:** 1 National Microbiology Laboratory at Guelph, Public Health Agency of Canada, Guelph, Ontario, Canada; 2 Department of Population Medicine, University of Guelph, Guelph, Ontario, Canada; McGill University Health Centre, CANADA

## Abstract

The global primary literature on Zika virus (ZIKV) (n = 233 studies and reports, up to March 1, 2016) has been compiled using a scoping review methodology to systematically identify and characterise the literature underpinning this broad topic using methods that are documented, updateable and reproducible. Our results indicate that more than half the primary literature on ZIKV has been published since 2011. The articles mainly covered three topic categories: epidemiology of ZIKV (surveillance and outbreak investigations) 56.6% (132/233), pathogenesis of ZIKV (case symptoms/ outcomes and diagnosis) 38.2% (89/233) and ZIKV studies (molecular characterisation and in vitro evaluation of the virus) 18.5% (43/233). There has been little reported in the primary literature on ZIKV vectors (12/233), surveillance for ZIKV (13/233), diagnostic tests (12/233) and transmission (10/233). Three papers reported on ZIKV prevention/control strategies, one investigated knowledge and attitudes of health professionals and two vector mapping studies were reported. The majority of studies used observational study designs, 89.7% (209/233), of which 62/233 were case studies or case series, while fewer (24/233) used experimental study designs. Several knowledge gaps were identified by this review with respect to ZIKV epidemiology, the importance of potential non-human primates and other hosts in the transmission cycle, the burden of disease in humans, and complications related to human infection with ZIKV. Historically there has been little research on ZIKV; however, given its current spread through Australasia and the Americas, research resources are now being allocated to close many of the knowledge gaps identified in this scoping review. Future updates of this project will probably demonstrate enhanced evidence and understanding of ZIKV and its impact on public health.

## Introduction

Zika virus (ZIKV) was first identified in rhesus monkeys in the Zika forest of Uganda in 1947 and has circulated in Africa and Asia relatively unnoticed for sixty years [1]. It is a Flavivirus transmitted by *Aedes* spp. mosquitoes, particularly *Aedes aegypti*, and infection is frequently asymptomatic. Clinical manifestations include rash, mild fever, arthralgia, conjunctivitis, myalgia, retro-orbital pain, headache and cutaneous maculopapular rash [2]. The epidemiology of ZIKV changed in 2007 when an outbreak occurred on Yap Island of the Federated States of Micronesia; this was the first report of infection outside of Africa or Asia. In 2013–2014 outbreaks occurred in New Caledonia, French Polynesia, the Cook Islands, Easter Island, Vanuatu, Samoa, Brazil (2015) and currently (March 2016) 31 countries in the Americas have reported autochthonous transmission [3]. With the geographic spread, a previously unreported clinical pattern began to emerge with an increase in cases of Guillain-Barré syndrome (French Polynesia) and a rise in infants born with microcephaly in Brazil [4].

A systematic summary of the global scientific knowledge regarding ZIKV and effective prevention and control measures is required to support evidence-informed decision making concerning this emerging public health issue. Scoping reviews are a synthesis method designed to address broad, often policy-driven research questions [5–7] by identifying all the relevant evidence concerning the issue and producing summaries of the findings [5,6,8]. Scoping reviews, similar to systematic reviews, follow a structured protocol for the identification and characterisation of the literature in a manner that is both reproducible and updateable [5,6,9]. Unlike a systematic review, a scoping review is well-suited to the identification of evidence on a broad topic, but does not include a quality assessment or in depth data extraction stage that would be required for meta-analysis of studies. Systematic reviews may be prioritized as a next step based on the results of a scoping review if adequate research exists on specific questions of interest [9,10]. Another important output of a scoping review is the identification of where evidence is lacking or non-existent to help direct future research and use of resources.

### Objective

In response to the current ZIKV outbreaks and changes in its epidemiology, a scoping review was conducted to capture all published literature addressing the following aspects of ZIKV: 1) ZIKV infection in humans, any host or vector—pathogenesis, epidemiology, diagnosis, conditions for virus transmission, and surveillance for ZIKV, 2) studies on ZIKV—pathogenesis, transmission and molecular mechanisms, 3) prevention strategies to prevent ZIKV infections and/or control of ZIKV harbouring vectors, and 4) societal knowledge, perception and attitudes towards ZIKV.

## Methods

### Review protocol, team and expertise

To ensure the scoping review methods were reproducible, transparent and consistent, a scoping review protocol was developed *a priori*. The protocol, list of definitions, search algorithms, abstract screening form and data characterization and utility form (DCU) are available upon request. The location of the repository of relevant articles and the dataset resulting from this review is available upon request.

The review team consisted of individuals with multi-disciplinary expertise in epidemiology, microbiology, veterinary public health, knowledge synthesis and information science.

### Review question and scope

This scoping review was conducted to answer the following question: What is the current state of the evidence on ZIKV pathogenesis, epidemiology, risk factors, diagnosis, surveillance methods, prevention and control strategies and, knowledge, societal attitudes and perceptions towards infections in humans, mosquito vectors and animal reservoirs?

### Search strategy

To ensure the search was comprehensive the term “zika” was implemented in the following bibliographic databases on January 27 2016 and updated March 1, 2016: Scopus, PubMed/MEDLINE, Embase, CINAHL (Cumulative Index to Nursing & Allied Health), CAB, LILACS (South American), Agricola and the COCHRANE library for any relevant trials in the trial registry. No limits were placed on the search and it was pretested in Scopus.

The capacity of the electronic search to identify all relevant primary research was verified by hand searching the reference lists of three ZIKV risk assessments, 19 literature reviews. Reference lists of the 3 risk assessments [11–13] and 19 selected relevant literature reviews were evaluated for additional research that had been omitted by the bibliographic database search [14–29]. From this exercise 34 grey literature reports with primary information and peer-reviewed primary literature articles were identified and added to the scoping review process.

Additional grey literature was identified by hand-searching the websites of the World Health Organisation (http://www.who.int/csr/disease/zika/en/), Pan American Health Organisation (http://www.paho.org/hq/index.php?option=com_content&view=article&id=11585&Itemid=41688&lang=en), Center for Disease Control and Prevention (http://www.cdc.gov/zika/index.html), Morbidity and Mortality Weekly Report (http://www.cdc.gov/mmwr/zika_reports.html), European Center for Disease Control (http://ecdc.europa.eu/en/healthtopics/zika_virus_infection/Pages/index.aspx) and ProMed-mail (http://www.promedmail.org/) for primary research reports, guidelines, epidemiological alerts, situation reports, surveillance bulletins and referenced publications that were not already captured. One hundred and eleven additional references were added to the project, many of these were guidelines / government reports and new articles that have not been indexed in the bibliographic databases yet.

### Relevance screening and inclusion criteria

A screening form was developed *a priori* to screen abstracts, titles and keywords of identified citations. Primary peer-reviewed articles were considered relevant if they addressed one or more aspects of the research question, conducted anywhere and anytime. Presently only articles in English and French are included but articles in Spanish and Portuguese are identified and can be included when resources are available. Primary research was defined as original research where authors generated and reported their own data.

### Study Characterisation and extraction

Complete articles of potentially relevant citations were reviewed using a data characterization and utility (DCU) form consisting of potentially 52 questions designed *a priori* to confirm article relevance, data utility and extraction of the main characteristics including reported information, study methodology, populations, intervention strategies, sampling, laboratory tests and outcome characteristics.

### Scoping review management, data charting and analysis

All potentially relevant citations identified by the literature search were imported into reference management software (RefWorks 2.0, ProQuest LLC, Bethesda, Maryland, USA), where duplicates were manually removed; the list of unique citations was then imported into a web-based electronic systematic review management platform (DistillerSR, Evidence Partners, Ottawa, Canada). All stages of the scoping review from relevance screening to data extraction were conducted within this software. Two reviewers independently completed all steps of the scoping review.

Data collected in the DCU form were exported into Excel spreadsheets (Microsoft Corporation, Redmond, WA), formatted, and analyzed descriptively (frequencies and percentages) to facilitate categorization, charting and discussion.

## Results

### Scoping review descriptive statistics

**Fig 1:**
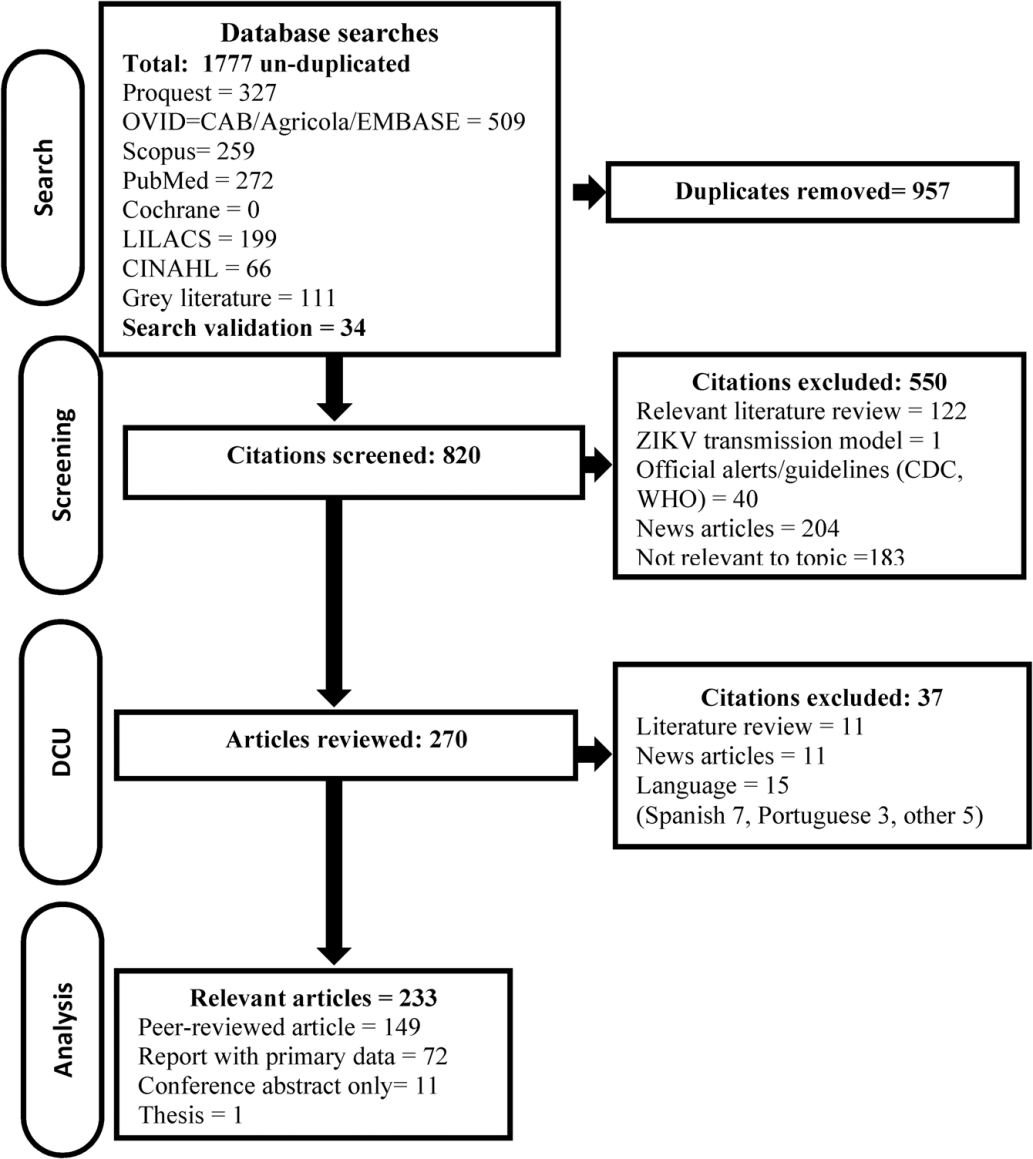
Flow of citations and articles through the scoping review process last updated March 1, 2016.

**Fig 2:**
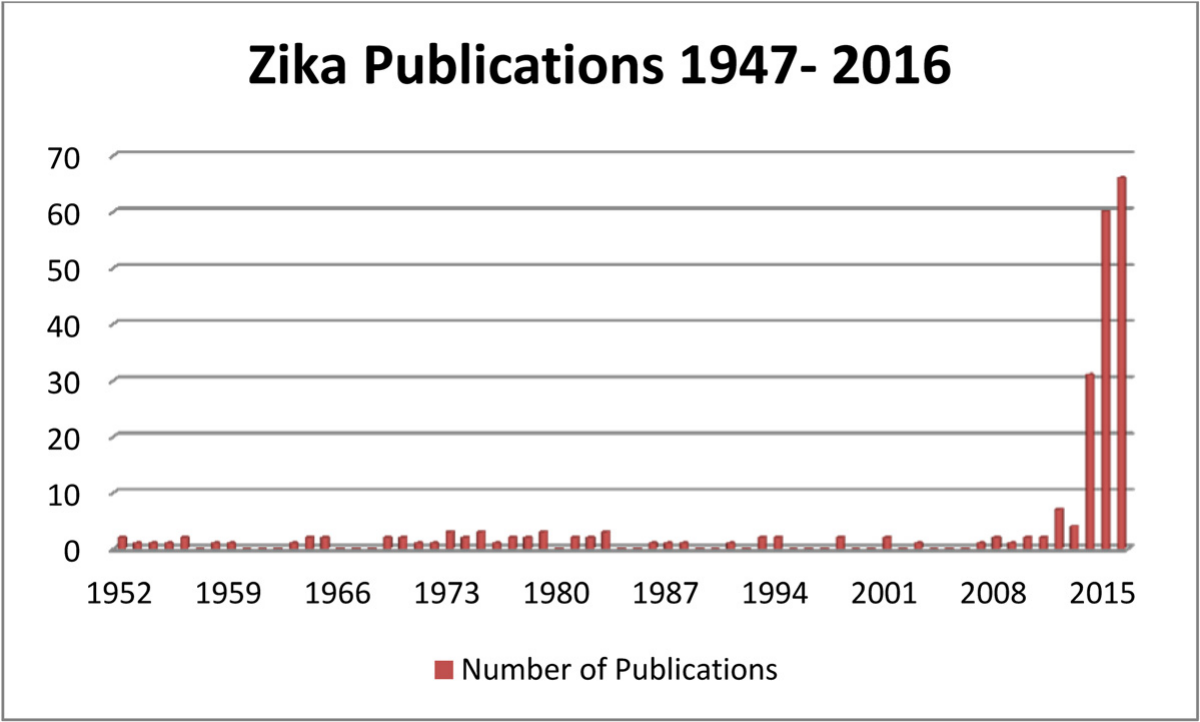
Number of primary literature publications on ZIKV by year of publication since its discovery in 1947 to search date March 1, 2016.

Of the 820 abstracts and titles screened for relevance, 270 were considered potentially relevant primary research/data and the full article was obtained, data was extracted and categorized for 233 relevant primary research articles in English or French, Fig 1. Research was scarce and sporadic after the virus was first isolated in 1947, until the outbreak on Yap Island, Micronesia in 2007; the majority of primary research on ZIKV has been published since 2011, 73.0% (170/233), Fig 2.

The general characteristics of the included articles are described in Table 1. Most included articles were peer-reviewed 63.9% (149/233) and in English 91.0% (212/233). Fifteen articles were in languages other than French and English (Spanish (7), Portuguese (3), Chinese (3), Russian (1), and German (1)) and Fig 1. The largest body of research is now from the Americas 34.87% (81/233), followed by Australasia 24.9% (58/233) and Africa, 24.0% (56/233), the remaining research was fairly evenly distributed over the other continents. The articles mainly covered three topic categories: epidemiology of ZIKV (surveillance and outbreak investigations) 56.6% (132/233), pathogenesis of ZIKV (case symptoms/ outcomes and diagnosis) 38.2% (89/233) and ZIKV studies (molecular characterisation and in vitro evaluation of the virus) 18.5% (43/233). There has been little reported in the primary literature on ZIKV vectors (12/233), ZIKV surveillance (13/233), diagnostic tests (12/233) and transmission (10/233). Three papers reported on ZIKV prevention/control strategies [30–32], one investigated knowledge and attitudes of health professionals [33] two vector mapping studies [34,35] and one ZIKV transmission model were reported [36]. The majority of studies used observational study designs, 89.7% (209/233) of which 62/233 were case studies or case series, while fewer (24/233) used experimental study designs.

**Table 1:** General characteristics of 233 included primary research publications.

Category		Count
**Type of citation**		
** **	Primary peer-reviewed	149
	Grey literature with primary data	72
	Conference proceeding	11
	Thesis	1
**Language**		
	English	212
** **	French	21
**Continent**		
	Central America/South America/Caribbean	64
** **	Australasia	58
** **	Africa	56
** **	Europe	25
** **	Asia	20
** **	North America	17
**Date of publication**		
** **	Pre 1960	11
** **	1960–1970	8
** **	1971–1980	18
** **	1981–1990	10
** **	1991–2000	7
** **	2001–2010	9
** **	2011-Present	170
**ZIKV topic category**		
	Epidemiology	132
** **	Pathogenesis	89
	Virus study	43
** **	Surveillance	13
** **	Diagnostic tests	12
** **	Vector study	12
** **	Transmission	10
** **	Mitigation	3
** **	Qualitative	1
** **	Other model	1
**Study design**		
** **	**Observational study**	**209**
	Outbreak investigation	67
** **	Case study or case-series	62
** **	Prevalence survey	48
** **	Surveillance or monitoring program	14
** **	Cross-sectional	7
** **	diagnostic test evaluation	7
	Molecular epidemiology	6
** **	Longitudinal study	1
** **	Case control	1
** **	**Experimental study**	**24**
** **	Challenge trial	22
** **	Controlled trial	2
	**Vector mapping model**	**2**

In the 233 included articles, human populations were the most frequently reported (191/233) with 15 studies focusing solely on pediatric populations (<16 years old), Table 2. Blood was the most common sample (179/191) used to evaluate ZIKV infection or seropositivity in humans, Table 2. Monkeys were the most frequently studied non-human hosts (7/13) followed by three recent studies (4 citations) on bats from which none report ZIKV isolation, Table 2. Mice were used for the majority of animal model experiments (13/15) while monkeys were used in 4/15 studies, Table 2.

**Table 2:** Human and non-human host populations studied in 233 included articles.

Category		Count	
**Humans**	** **	**191**	** **
	adults/ all ages	176	
	pediatric only	15	
** **	**human samples**		
	blood	179	
	urine	16	
	saliva	10	
	fetus / placenta	8	
	amniotic fluid	5	
	head circumference	4	
	cerebrospinal fluid	4	
	semen	2	
	breast milk	2	
	nasopharynx swab	2	
	conjunctivae swab	1	
**Host**	** **	**13**	** **
	bats	4	
	chicken/ duck (domestic)	1	
	domestic ruminants	1	
	horse	1	
	monkey	7	
	rodents	2	
	small mammals	1	
	wild birds	1	
**Animal model**	**experimental animals**	**15**	
	Mice	13	
	Non-human primates	4	Rhesus monkey
	Other animal species	2	cotton rats, guinea pigs, rabbits
**Vectors**	**45 mosquito species**	**26**	*See Table 4*

### ZIKV virus studies

A small number of studies examined the attributes of ZIKV, Table 3. Early reports describe a 40 mμ, spherical shaped virus [37] that infects the nucleus of cells [27]. Others examined the antigenic relatedness of various Flaviviruses [26,38]. The virus growth, replication and survival in human cell lines [39] and in vivo (mice) was investigated [40] and the pathogenesis in mice has also been described [38,41,42]. The evaluation of a mouse model for virus propagation is described in three studies [42–44]; the mouse model was also used in 13 studies to identify and propagate ZIKV. In one report, researchers describe the use of chick embryos and inoculation of the yolk sac for virus propagation as being comparable to intracerebral inoculation of mice [45]. Earlier studies were identified that examined the cross neutralization tests and cross complement fixation tests on a number of new and known viruses to examine their relatedness [45] and used a monkey model to show there was no cross-immunity with yellow-fever and ZIKV [46].

**Table 3:** 45 studies examined the ZIKV characteristics: genotype, molecular characterisation, pathogenic attributes and virus propagation in mice.

	Number of studies
**Genotype reported:**	
Asian lineage	33
East/Central/South African lineage (Uganda)	9
West African lineage (Senegal/Nigerian)	5
**Molecular characterisation:**	
Phylogeny was reported (e.g. dentogram, tree)	26
Virus was reported to be partially or fully sequenced	25
Evolution of the virus discussed	12
Examined proportion of nucleotide identities among strains and/or codon adaptation and fitness	5
**Pathogenic attributes:**	
Virus propagation in mice via intracerebral inoculation	4
Antigenic relatedness of Flaviviruses	2
Viral entry into cells	2
Virus morphology (ZIKV: 40 mµ, spherical in shape)	1
Presence of virus-specific antigen in the nuclei of Zika virus-infected cells (vertebrate & invertebrate)	1
Virus growth in vitro	1
Virus spread within mice	1
Virus propagation in chick embryos	1

ZIKV genotype was provided in 17.6% (41/233) of studies, 36 observational studies and 5 experimental studies, Table 3 [21,32,39,46–81]. One study failed to report the genotype even though sequencing was done [82]. Due to the outbreaks in Australasia since 2007 and more recently in the Americas, many of the studies reporting genotype reported the Asian type (33/41), Table 3. With respect to studies that lend evidence to the molecular characterisation of the virus, authors frequently reported the virus had been partially sequenced, provided a gene bank number or provided a phylogenic tree in the paper [21,47,50–55,57–63,66–81,83]. A number of studies discussed ZIKV genetic relatedness other Flaviviruses and the evolution of ZIKV [47,50–54,57–59,63,68,70,83]. In a few studies the evolution of the virus (12/41) was discussed or proportions of nucleotide identities across strains (5/41) were provided in the paper. A recent paper examined codon adaptation and fitness [51] and concluded that across studies no particular mutations or evidence of adaptation to a new vector would explain the recent spread through the Pacific Islands to the Americas.

### Zika Vectors

Known ZIKV vectors are all mosquitos; no evidence was identified for any other type of vector. Table 4 reports on the 26 mosquito vector studies that tested 45 different mosquito species for either a natural infection with ZIKV or evaluated the mosquito species for vector competence to transmit ZIKV. Eighteen species of mosquitos were found to be positive for ZIKV during epidemiological sampling in Africa and Asia from 1956 to 2015 and eight were evaluated experimentally for vector competence, Table 4. *Ae*. *aegypti* 15/26, and *Ae*. *africanus* 10/26 were investigated most often. *Ae*. *albopictus*, a species of particular interest as a possible vector for ZIKV in North America, was evaluated for vector competence in one study and as a naturally infected vector of ZIKV in two studies.

**Table 4:** Vector species evaluated in 26 studies for carriage of ZIKV or vector competence to transmit ZIKV.

Ref	Mosquito species	Number of Studies	Challenge trials	Naturally infected with ZIKV studies
	**Total Studies:**	26	10	12
[31,35,64,83–95]	*Aedes aegypti*	16	6	5
[31]	*Aedes aegypti formosus*	1	1	
[1,35,38,44,70,91,95–98]	*Aedes africanus*	10	2	4
[65,83,87]	*Aedes albopictus*	3	1	1
[97]	*Aedes apicoargenteus*	1		1
[93]	*Aedes argentepunctatus*	1		
[93]	*Aedes cozi*	1		
[93,94]	*Aedes cumminsi*	2		
[35,86,91,93,99]	*Aedes dalzielii*	5		4
[86]	*Aedes fowleri*	1		1
[35,86,91–94,96,99]	*Aedes furcifer*	8		4
[85,100]	*Aedes hensilii*	2	1	
[91]	*Aedes hirsutus*	1		1
[98]	*Aedes ingrami*	1		
[35,84,86,91,93–96,99]	*Aedes luteocephalus*	9	1	5
[35,91,94]	*Aedes metallicus*	3		1
[35,86]	*Aedes minutus*	2		1
[35,86,93]	*Aedes neoafricanus*	3		1
[70,93,94]	*Aedes opok*	3		1
[35]	*Aedes patas*	1		
[83,95]	*Aedes simpsoni*	2		
[35,86,91,93,99]	*Aedes taylori*	5		3
[84,91,93]	*Aedes unilineatus*	3	1	1
[85]	*Aedes vexans*	1		
[35,84,86,91–93,95,99]	*Aedes vittatus*	8	1	2
[35]	*Aedes coustani*	1		1
[91]	*Anopheles coustani*	1		
[35,83,94]	*Anopheles gambiae*	3		
[94]	*Anopheles nili*	1		
[35]	*Cercopithecus aethiops*	1		
[98]	*Chrysops centurionis*	1		
[98]	*Chrysops funebris*	1		
[85]	*Coquillettidia crassipes*	1		
[98]	*Culex annulioris*	1		
[85]	*Culex gossi*	1		
[85]	*Culex nigropunctatus*	1		
[91]	*Culex perfuscus*	1		1
[94]	*Culex poicilipes*	1		
[83,85]	*Culex quinquefasciatus*	2		
[85]	*Culex sitiens*	1		
[83]	*Eretmapodites quinquevittatus*	1		
[85]	*Lutzia fuscana*	1		
[83]	*Mansonia africana*	1		
[98]	*Mansonia aurites*	1		
[35,83,91,99]	*Mansonia uniformis*	4		2

Experimental studies examining vector competence and feeding activity under laboratory conditions (24–29 C and 75–95% relative humidity) mainly focused on *Ae*. *aegypti* and provided data on infection rates, dissemination rates, transmission rates, minimum infection rate, entomologic inoculation rate or mean biting rate, Table 5. Studies on *Ae*. *aegypti* demonstrated that individual mosquitos held under laboratory conditions transmitted ZIKV 33–100% of the time and transmission of the virus occurred irrespective of whether the mosquito was engorged with a blood meal [90]. In one experiment infected *Ae*. *aegypti* transmitted ZIKV to a rhesus monkey 72 days post mosquito infection [89]. Mitigation by biological control was investigated in one experiment where the researcher examined the transferability of mosquito resistance to ZIKV between species (*Ae*. *aegypti* and *Ae*. *formosus*), however they concluded the resistance gene is complex and not easily manipulated [31].

**Table 5:** Summary of eight studies on vector competence and behaviour.

Ref	Country	Year study conducted	Mosquito Species	Infection rate	Dissemination rate						Transmission rate				Proportion infected	Proportion disseminated ZIKV	MIR	EIR	MBR
	Africa				3dpi	5 dpi	8 dpi	10 dpi	15 dpi	20–60 dpi	4 dpi	7 dpi	10 dpi	15 dpi					
[84]	Senegal	2015	*Ae*. *aegypti*	highest 10dpi		0%		0–10%	0–50%						55%	5.80%			
			*Ae*. *unilineatus*	highest 10dpi		0–10%									18.70%	5.30%			
			*Ae*. *vittatus*	highest 10dpi		0%		0–100%	0–100%					20–50%	14.40%	27%			
			*Ae*. *luteocephalus*	highest 15dpi		0%			0–100%					20–50%	75%	42.20%			
[91]	Senegal	2011/04–2011/12	*Ae*. *aegypti*, *Ae*. *africanus*, *Ae*. *dalzielii*, *Ae*. *furcifer*, *Ae*. *vittatus*, *Cx*. *perfuscus*, *Ae*. *taylori*, *Ae*. *hirsutus* and *Ae*. *metallicus*, *Ae*. *unilineatus*, *Ma*. *Unif*, *An*. *coustani*, *An*. *andoustani*														3–10	0.005–0.052	0.48 to 12.78
[90]	Senegal	1976	*Ae*. *aegypti*									88%	79–100% (7–30 dpi)/ titer 2–6						
[89]	Nigeria	1956	*Ae*. *aegypti*						10 ^3.4^	10^4.7 to 5.6^									
	**Asia**																		
[65]	Singapore	2013	*Ae*. *albopictus*	100% at 3dpi	25%+	50%+	100% +	100%+	100%+		first isolate+		100%+						
[64]	Singapore	2012	*Ae*. *aegypti*	87.5% at 3 dpi, 100% at 6 dpi	first obs 4 dpi +	62%+		100%+	100%+										
[85,100]	Yap island	2007/04–2007/07	*Ae*. *(Stegomyia) hensilii*	7.1% (4.9 titre*)		0% (4.9% titer*)													
** **				80% (5.7 titer*)		12.5% (5.7 titer*)													
** **				86.1% (5.9 titre*)		22.5% (5.9 titer*)													

* log10 pfu/ml, environment "humid" and 28C, dpi = days post infection,

+ dissemination rate = divide the number of infected salivary glands by the total number of mosquitoes with infected midgets. Transmission rate = divide the number of positive saliva by the number of infected salivary glands. MIR = Minimum infection rate, estimated number of positive mosquitoes per 1000 mosquitoes tested. EIR = Entomologic inoculation rate, number of infected mosquito bites per person, per evening. MBR = mean biting rate, number of female mosquitoes captured per person per evening

Active mosquito surveillance programs were reported in three studies in Uganda, Senegal and Burkina Faso between 1946 and 1986, where routine samples were collected [35,94,97]. Studies that conducted surveillance activities or prevalence surveys of mosquitos for ZIKV were identified from Africa and Asia and several species were implicated as possible vectors including *Ae*. *aegypti* (5 studies) and *Ae*. *albopictus* (1 study), Table 6. The results of the studies in Table 6 were not necessarily conducted when ZIKV was actively circulating.

**Table 6:** Twelve studies from Africa and Asia between 1956 and 2011 that sampled mosquitos for ZIKV. Studies were conducted as prevalence surveys or part of entomological surveillance for arboviruses.

Ref	Country	Date / place of sampling	ZIKV positive mosquito species	Positive/N	Other epidemiological information	Other mosquito species tested
Africa						
[94]	Burkina Faso	1984/09–1984/11: area not specified	*Ae*. *furcifer*, *Ae*. *luteocephalus*, *Ae*. *opok*	9/ 1853 pools	Based on entomological arbovirus surveillance 1983–1986.	*Ae*. *aegypti*, *Cx*. *poicilipes*, *Ae*.*metallicus*, *Ae*. *cumminsi*, *Anopheles gambiae*, *An*. *nili*
[92]	Côte d’Ivoire	1999: area not specified	*Ae*. *vittatus*, *Ae*. *furcifer*, *Ae*. *aegypti*.	3/159 pools		
[83]	Gabon	2007: Libreville suburbs	*Ae*. *albopictus*	2/247 pools	First isolation of ZIKV in Gabon in 2007 during a simultaneous outbreak of Chikungunya and Dengue.	*Ae*. *aegypti*, *Cx quinquefasciatus*, *Ae*. *simpsoni complex*, *An*. *gambiae*, *M*. *africana*, *M*. *uniformis*, *Cx*. *quinquefasciatus*, *Eretmapodites quinquevittatus*
[95]	Nigeria	1969: Jos Plateau	*Ae*. *luteocephalus*	2/205 pools		*Ae*. *aegypti*, *Ae*. *africanus*, *Ae*. *vittatus*, *Ae*. *simpsoni*
[91]	Senegal	2011/06–2011/12: Kedougou area	*Ae*. *luteocephalus* (5 pools), *Ae*. *africanus* (5), *Ae*. *vittatus* (3), *Ae*. *taylori* (2), *Ae*. *dalzielii* (2), *Ae*. *hirsutu*s (2) and *Ae*. *metallicus* (2), *Ae*. *aegypti* (1), *Ae*. *unilineatus* (1), *M*. *uniformis* (1), *Cx*. *perfuscus* (1), *An*. *coustan*i (1)	31/1700 pools	Thirty-one ZIKV infected pools: June (9.7%), September (32.2%), October (35.5%), November (19.3%) and December (3.2%)	*An*. *coustani*, *Ae*. *andoustani*, *Ae*. *furcifer*
[93]	Senegal	1990: Senegal	*Ae*. *aegypti*, *Ae*. *dalzielii*, *Ae*. *luteocephalus*	3/151 pools		*Ae*. *taylor*, *Ae*. *unilineatus*, *Ae*. *cozi*, *Ae*. *neoafricanus*, *Ae*. *opok*, *Ae*. *cumminsii*, *Ae*. *argentepunctatus*, *Ae*. *furcifer*, *Ae*. *vittatus*
[86]	Senegal	1988: Southern Senegal	*Ae*. *furcifer (25/62 pools)*, *Ae*. *aegypti*, *Ae*. *dalzielii*, *Ae*. *taylori*, *Ae*. *neoafricanus*, *Ae*. *fowleri*, *Ae*. *minutus*	27/435 pools	ZIKV seemed to have an epizootic annual cycle. In 16 ZIKV positive pools, dengue-2 was also isolated.	
		1989: Southern Senegal		29/654 pools		
		1990: Southern Senegal		3/497 pools		
		1991: Southern Senegal		3/1264 pools		
[99]	Senegal	1972: area not specified	*Ae*. *dalzielii*, *Ae*. *furcifer*, *Ae*. *luteocephalus*, *Ae*. *vittatus*, *Ae*. *taylori*, *M*. *uniformis*	2/204	130,000 mosquitos from 69 different species were examined 1972–77.	
		1973: area not specified		16/798		
		1976: area not specified		19/599 pools		
[38]	Uganda	1956/05–1956/08: Lunya forest	*Ae*. *africanus*	2/11 pools	11 pools = 1355 mosquitos	
[98]	Uganda	1961/11–1963/06: Zika forest	*Ae*. *africanus*	13/688 pools	Positive pools occurred 1962/05-1962/09 and 1962/11.	*M*. *aurites*, *C*. *centurionis*, *C*. *funebris*, *C*. *annulioris*, *A*. *ingrami*
[97]	Uganda	1969–1970: Zika Forest	*Ae*. *africanus* (14 isolates), *Ae*. *apicoargenteus* (1)	15/105 pools	No ZIKV isolation for 6 years and 73 days (since 1962/11): 8 isolations 1969/04–1969/06 and a 192 day lapse before 7 isolations 1970/04–1970/08.	
Asia						
[87]	Malaysia	1966: Bentong	*Ae*. *aegypti*	1/296 pools		(pools- mosquitos) *Ae*. *aegypti* (58–1,277), *Ae*. *albopictus* (59–4492), 23 other *Aedes* species (179–27636)

Several epidemiological studies examining risk factors for mosquito abundance, human exposure to mosquitos and conditions for ZIKV infected mosquitos were identified by the scoping review. Observations include some mosquito species such as *Ae*. *aegypti* are well suited to urban environments, are often trapped within homes [87] and are able to reproduce in water containers which are frequently found in homes. For example, on Yap Island during the 2007 outbreak 87% of the homes were found to have mosquito infested water containers [85,100]. Biting behaviour of mosquitos was shown to increase in the two months following rainfall and decreased at temperatures less than 13°C [97]. A study conducted in Senegal found the forest canopy and forest ground areas were more likely to yield ZIKV positive mosquitoes [91]. From 13 species of mosquitos examined, *Ae*. *furcifer* showed significant temporal variation and *Ae*. *luteocephalus* and *Ae*. *taylori* showed a significant correlation between biting and infection rates with a one month lag time [91].

A vector mapping model based on mosquito data collected in Senegal between 1972 and 2008 demonstrated ZIKV was not correlated with the incidence of other viruses, the abundance of mosquitos, mean temperature or humidity [35]. However, ZIKV was shown to increase 12% (95% CI = 5%-21%) for each additional inch of rain above the baseline, was positively correlated with Chikungunya virus at a 6 year lag time, and was correlated with *Ae*. *fuciferi* and *Ae*. *taylori* at a 13 year lag time [35]. The minimum infection rate for *Ae*. *furcifer* and *Ae*. *taylori* was 80% (95% CI = 41%-94%) lower than for *Ae*. *luteocephalus* [35].

### Non-human hosts of ZIKV

Zika virus was first isolated in 1947 in a rhesus sentinel monkey used for yellow fever surveillance [1]. Subsequent studies in Africa to evaluate potential hosts of ZIKV examined a number of small mammals in the Zika forest and bats in Uganda, but failed to identify ZIKV or seroreactivity to ZIKV in any sample, Table 7 [98,101]. Surveys of various monkeys in Uganda, Gabon and Burkina Faso showed a range of seroprevalence for ZIKV from 0% to >50% in some species, Table 7 [97,102–104]. Surveys from Indonesia (1978) and Pakistan (1983) sampled a number of rodents, bats, wild birds and domestic livestock; they reported some ZIKV seropositivity in rodents, bats, ducks, horses and large domestic livestock [28,105]. One study, published in two papers, on the orangutans in the Sabah region of Malaysia reported a higher seroprevalence among free-ranging orangutans compared to the semi-captive group and higher seroprevalence among humans than orangutans [22,23]. They hypothesize that ZIKV among orangutans may be incidental infections from mosquitos that had contact with viremic humans or another sylvatic cycle [22,23]. At this time it has been experimentally demonstrated that infected mosquitos can transmit ZIKV to mice and monkeys under controlled laboratory conditions [89]. However, no studies were identified that examined the relative importance of non-human primates or other potential hosts compared to humans in the transmission cycle of ZIKV.

**Table 7:** Potential host populations sampled for ZIKV in nine studies conducted between 1952 and 2016.

Ref	Country	Date / place of sampling	Population sampled (positive/N)
**Africa **		** **	** **
[104]	Burkina Faso	1983–1984	Monkey, not specified (0/9)
[103]	Gabon	1979–1980:	Monkey, Simian (9/34)
[101]	Uganda	2009–2013: area not described	Bats (0/1067)
[106]	Uganda, Kenya, Democratic Republic of Congo	2011–2012	Bats (conference proceeding, no data)
[107]	Uganda	after 2011	Bats (conference proceeding, no data)
[102]	Uganda	1972: Entebbe area	Monkey (9/21): grey vervet and red tail
[97]	Uganda	1969–1970: Around Kisubi forest and Bwame forest	Monkeys: vervet monkey (23/52), redtail monkey (4/21), mona monkey (0/1), black mangabey (2/4), lowland colobus (5/11), others (7/16)
[98]	Uganda	1962–1963: Zika forest	(0/25) "small mammals": 1 potto, 3 palm civets, 4 squirrels, 2 tree rats, 7 giant pouched rats, 8 field rats
[1]	Uganda	1947/04: Zika forest	(1/1) Rhesus sentinel monkey: first identification of ZIKV
**Asia**			
[28]	Indonesia	1978: Lombok	Horse (3/15), cow (4/41), carabao (1/13), goat (7/35), chicken (0/78), duck (2/52), bat (6/71), rat (0/25), wild bird (0/17)
[22] [23]	Malaysia	1996–1998: Sabah region	Orangutans semi- captive (1/31) and free-ranging (5/40)
[105]	Pakistan	1983: Pakistan	Rodents: *Tatera indicia* (3/47), *Meriones hurrianae* (2/33), *Bandicota bengalensis* (1/2) cow (0/45), buffalo (0/33), sheep (1/46), goat (1/48)

Pathogenesis of ZIKV infection in rhesus monkeys has been described for two animals; the initial sentinel monkey developed fever and was viremic on day 3 of the fever. The second monkey who was subcutaneously challenged with ZIKV showed no signs of disease, but was viremic on days 4–5 and tested seropositive 30 days after exposure [1,44]. Mice were primarily used across 13 laboratory studies published between 1952 and 1976 to study pathogenesis, transmission and to propagate ZIKV, Table 8 [1,38,42,44]. Pathogenesis of ZIKV infection in mice mainly consists of degeneration of nerve cells, wide-spread softening of the brain and often porencephaly [38,42], with little effect on other major organs [38]. In one experiment challenged rats, guinea pigs and rabbits did not show signs of illness, however, the rabbits became seropositive while the guinea pigs died [38]. No ZIKV could be identified in the guinea pigs to indicate the virus had a role in the deaths [38].

**Table 8:** List of studies that used an animal model to propagate ZIKV or investigate the pathology and transmission of ZIKV in mice, monkeys or other laboratory animals.

Ref	Country	Year	Study objective	Animal model
[40]	England	1976	Pathogenesis	mice
[42]	England	1971	Pathogenesis	mice
[46]	Uganda	1970	ZIKV cross-immunity with yellow fever	rhesus and vervet monkeys
[87]	Malaysia	1969	ZIKV propagation	mice
[98]	Uganda	1964	ZIKV propagation	mice
[108]	Malaysia, Thailand and N. Vietnam.	1963	ZIKV propagation	mice
[38]	Uganda	1958	Histopathogenesis	mice
[89]	Nigeria	1956	ZIKV propagation	mice
[88]	Nigeria	1956	Transmission	mice
[41]	United States	1955	Histopathogenesis	mice
[43]	Nigeria	1954	ZIKV propagation	mice
[37]	United States	1953	ZIKV propagation	mice
[1]	Uganda	1952	ZIKV propagation	mice
			Transmission	Rhesus monkey
[44]	Uganda	1952	ZIKV propagation	mice
			Isolation	rhesus monkey
			Pathogenesis	cotton rats, guinea pigs, rabbits
[45]	Uganda and Tanzania	1951	ZIKV propagation	chick embryo

### ZIKV in humans

#### Epidemiology

Studies investigating the epidemiology of ZIKV in humans (120/233) provided prevalence estimates (46) or summarized an outbreak situation (74) from 1952–2016 and included the following healthy populations: general and pediatric populations, blood donors and military personnel and acute viral fever patients as indicated in Table 9. The majority of the prevalence surveys and outbreak reports were from the Americas (44), Asia/Australasia (42) and Africa (30); some reported for more than one region. Risk factors for testing ZIKV positive were examined in three studies. On Yap Island, a study found men 77% [95% CI, 72% to 83%] were more likely to have IgM antibodies against ZIKV virus compared to women 68% [95% CI, 62% to 74%], which was a relative risk of 1.1 [95% CI, 1.0 to 1.2] [100]. Higher density housing was significantly associated with increased risk of arbovirus infections in Kenya (1970) [109]. A recent study from Zambia found that indoor residual spraying had an adjusted odds ratio of 0.81, 95% CI [0.66, 0.99] and having an iron sheet roof on the home was protective against being ZIKV seropositive. However, increasing age, farming and having a grass roof significantly increased the risk of being ZIKV seropositive [110].

**Table 9:** One hundred and twenty prevalence studies and outbreak reports that report ZIKV infection in human populations published between 1952 and 2016.

Ref	Country	Date / place of sampling	Population sampled	Measure-ment	Prevalence/ Rate	Positive / N	Comment
**Africa**	** **	** **	** **	** **	** **	** **	** **
[111]	Angola	1960: several regions	General population- adults	Prevalence	27.0%	133/492	(20/42 Neutralization test positive)
[112]	Burundi	1980–1982: many areas	General population- all ages	Prevalence	1.4%	9/623	
[113]	Cameroon	2010: Buea and Tiko in the Fako division (80% of the population)	All ages- hospital submissions	Prevalence	38.0%	30/79	
[114]	Cameroon	1972: several areas	General population- all ages	Prevalence	7.0%	83/1186	
[115]	Cape Verde	2015/09/27–2015/12/06	General population- all ages	Outbreak		4744 cases	165 (sept 27-Oct14) and 4744 (Sept 27- Dec 6) suspected cases. (17 confirmed)
[116]	Cape Verde	2015/10–2016/01/17	General population- all ages	Outbreak		7081 cases	
[117]	Cape Verde	2015/10–2016/01/31	General population- all ages	Outbreak		7258 cases	
[118]	Central African Republic	1979: M'Bomou region (population of 30 000)	General population- adults	Prevalence	59.0%	271/459	Prevalence: Bangassou 47%/111, M'Ballazime 62.4%/125, Iongofongo 68.5%/162, Ouango 47.5%/61
[92]	Côte d’Ivoire	1999/08: Kakpin	Adult	Prevalence	54.2%	13/24	
[119]	Ethiopia	2013: Dire Dawa town	All ages- hospital submissions	Prevalence	10.0%	5/50	
[83]	Gabon	2007–2010: entire country	All ages- hospital submissions	Prevalence	0.1%	4/4312	
[103]	Gabon	1979/10–1980/03: Franceville	General population- adults	Prevalence	14.7%	29/197	
[120]	Gabon	1975: South East Gabon	General population- adults	Prevalence		?/1276	ZIKV positive number not specified
[121]	Guinea	2010: N'Zerekore and Faranah	All ages- hospital submissions	Prevalence			?/151, number ZIKV positive not reported.
[122,123]	Kenya	2013: Kenya	General population- all ages	Prevalence	0.0%	0/351	
[124]	Kenya	1969: Ahero	Paediatric- school age children	Prevalence	7.2%	40/559	
[109]	Kenya	1966–1968: Central Nyanza, Kitui district, Malindi district	General population- all ages	Prevalence	17.6%	475/2698	Prevalence: Central Nyanza 3.3%, Kitui district 1.3%, Malindi district 52%
[125]	Nigeria	1980: near Kainji Dam	General population- all ages	Prevalence	56.2%	150/267	
[126]	Nigeria	1975: Igbo-Ora	General population- all ages	Prevalence	20.0%	4/20	1 virus isolation
[127]	Nigeria	1971–1974: Oshogbo, Igbo-Ora, Ibadan and Oyo.	General population- pediatric	Prevalence	27.0%	51/189	2 ZIKV isolations /10778 samples
[128]	Nigeria	1965: Benue Plateau State	General population- all ages	Prevalence	21.4%	15/70	
[128]	Nigeria	1970–1971: Benue Plateau State	General population- all ages	Prevalence	12.2%	18/147	Samples taken during a yellow fever outbreak 1970
[129]	Nigeria	1964–1970: whole country	Paediatric- hospital submissions	Prevalence	0.0%	3/12613	3 ZIKV isolates were from 1968/ from 171 isolated arboviruses
[43]	Nigeria	1952: Uburu	General population- adults	Prevalence	59.5%	50/84	
[130]	Nigeria	1955: Ilobi	all ages	Prevalence	55.1%	114/207	
[130]	Nigeria	1951: Ilaro	all ages	Prevalence	44.3%	43/97	
[131]	Senegal	2009/07–2013/03: eastern Senegal (population:141 226)	Adults- clinic submissions	prevalence	0.1%	9/13845	All 9 IgM ZIKV positive cases were in 2011 only.
[86]	Senegal	1990: Southern Senegal	General population- adults	Prevalence	2.8%	11/396	
[86]	Senegal	1988: Southern Senegal	General population- adults	Prevalence	10.1%	46/456	
[132]	Senegal	1972–5: several areas	General population- all ages	Prevalence	58.3%	1432/2457	
[133]	Sierra Leone	1972: several areas	Paediatric	Prevalence	6.9%	62/899	
[44]	Uganda	1952 Zika forest, Kampala, Bwamba, West Nile areas	General population- all ages	Prevalence	6.1%	6/99	Prevalence: Zika forest 0%, Kampala 0%, Bwamba 20%, West Nile 9.5%.
[45]	Uganda and Tanzania	1951: not reported	General population- all ages	Prevalence		?/127	Order of prevalence: Bwamba > Ntaya > ZIKV > Uganda S > West Nile > Bunyamwera > Semliki Forest viruses
[110]	Zambia	2010: Western and North-Western province	General population- all ages	Prevalence	6.0%	217/3625	
**Asia / Australasia**							
[134–136]	American Samoa	up to 2016-02-07	General population- all ages	Outbreak			551 cases (2 confirmed)
[137–142]	Cook Islands	2014	General population- all ages	Outbreak			932 cases (18 confirmed and 50/80 in Tahiti confirmed.)
[79,138]	Easter Island (Chile)	2014/01–2014/06: Easter Island	General population- all ages	Outbreak prevalence	2.3%	89/3860	Easter Island population = 3860
[143]	Fiji	2014/08/	General population- all ages	Outbreak		.	2/6 suspect cases were ZIKV confirmed
[72,116, 139,144–148]	French Polynesia	2013/10/30–2014/04	General population- all ages	Outbreak Attack rate	12% (10–40 depending on the island)	32000/268207	8750 suspected cases. (383–460 confirmed) Estimate: 32000 cases, 11% of the population 268, 207 on 67 islands
[144]	French Polynesia	2014/02/04–2014/03/13	General population- all ages	Prevalence	41.3%		Estimated that 50% of people were asymptomatic
[137]	French Polynesia	2013–2014: French Polynesia	General population- all ages	Outbreak			8746 cases (>30000 medical consultations)
[71,147]	French Polynesia	2013/11–2014/02: French Polynesia	Adult- blood donors	Outbreak prevalence	2.8%	42/1505	11/42 reported ZIKV like illness 3 to 10 days after donation
[149]	French Polynesia	2011/07–2013/10: French Polynesia	Adults- blood donors	Prevalence	0.8%	5/593	
[53]	Indonesia	2014/12-2015/04	All ages- hospital submissions dengue negative	Prevalence	1%	1/103	
[150]	Indonesia	2004–2005: Bandung, West Java	All ages- hospital submissions dengue negative	Prevalence			?/95, number ZIKV positive not reported.
[28]	Indonesia	1978: Lombok	General population- all ages	Prevalence	2.0%	9/446	
[151]	Indonesia	1973: Tamampu, Malili, Balikpapan (South Sulawesi and East Kalimantan)	General population- all ages	Prevalence	9.5%	21/222	Prevalence: 27/160 Timampu, 24/40 Malili, 20/22 Balikpapan
[23]	Malaysia	1996–1997: Borneo	General population- all ages	Prevalence	44.1%	9/30 native & 40/81 immigrant	
[108]	Malaysia	1953–4: Kuala Lumpur	General population- all ages	Prevalence	75.0%	75/100	
[135]	Marshall Islands	2016/02/14	General population- all ages	Outbreak			First confirmed case. 1/6 suspect cases positive ZIKV
[137–139,142, 152,153]	New Caledonia	2013/11–2014/07	General population- all ages	Outbreak			1400 cases
[105]	Pakistan	1983	Adult	Prevalence	2.3%	1/43	
[154–156]	Samoa	2015/09–2015/12/06	Gen pop- all ages	Outbreak			3/40 suspect cases were confirmed.
[140,157–165]	Solomon Islands	2015/02–2015/05/24	General population- all ages	Outbreak			310 cases (5 confirmed)
[166]	Thailand	2015: Northern Thailand	Adult	Prevalence	80%	16/21	16/21 showed sero-reactivity to ZIKV, only 2 exclusively to ZIKV
[108]	Thailand	1954: Thailand	Adult	Prevalence	16.0%	8/25 south & 0/25 north	
[135, 167,168]	Tonga	2016/01–2016/02/14	gen pop- all ages	outbreak		.	> 800 suspect cases and 2 confirmed
[157,162]	Vanuatu	2015/04/26	General population- all ages	Outbreak			1st confirmed case
[108]	Vietnam	1954: North Vietnam, River Delta of Tomkin area	General population- all ages	Prevalence	4.0%	2/50	
[100, 147]	Yap Island, Micronesia	2007/04/01–2007/08/09	General population- all ages	Outbreak prevalence	74.3%	414/557 sero-prevalence	Yap Island had a population of 7391 at time of outbreak RT-PCR positive
				Outbreak prevalence	14.5%	185/1276	
				attack rate	14.6/1000 persons		Ranged 3.6–21.5/1000 by community
				Infection rate	73% (95% CI, 68 to 77)		
				Clinical symptoms	18% (95% CI, 10 to 27)	919 (95% CI, 480 to 1357)/6892	
**South and Central America**							
[169]	Barbados	2016/01/14-15	General population- all ages	Outbreak			3 cases: autochthonous transmission 2016/01
[117,170]	Brazil	2015/05–2015/02	General population- all ages	Outbreak			497593–1 482 701 cases estimated (they stopped counting)
[4,171, 172]	Brazil	2015/05-2015/12/01	General population- all ages	Outbreak			3 fatalities: One man with co-morbidities, one healthy 20 year old woman and an infant.
[173]	Brazil	2015/02–2015/06: Salvador Brazil	General population- all ages	Outbreak attack rate	5.5 cases/1000 persons		14835 cases (not confirmed)
[174]	Bolivia	2016-01-08	General population- all ages	Outbreak			1 case: autochthonous transmission 2016/01
[117,170,175]	Colombia	2015/10/31–2016/02/06	General population- all ages	Outbreak			31 555 cases (1504 confirmed)
[116]	Colombia	2015/10/31–2016/01/23	General population- all ages	Outbreak			20297 cases
[176]	Colombia	2015/09/22–2016/01/02	General population- all ages	Outbreak			11712 cases (746 confirmed) and 1 fatality: sickle cell disease (previously associated with severe dengue symptoms)
[4,54, 171]	Colombia	2015/10/31–2015/12/01	All ages	Outbreak			autochthonous transmission 9/22 confirmed cases Sincelejo area
[177]	Dominican Republic	2016/01/23	General population- all ages	Outbreak			10 cases; autochthonous transmission 2016/01
[169]	Ecuador	2016/01/14-15	General population- all ages	Outbreak			2 cases; autochthonous transmission 2016/01
[116,178]	El Salvador	2015/11–2015/12/31	General population- all ages	Outbreak			3836 suspect cases, 46 hospitalizations and 1 death; patient had many co-morbidities
[4]	El Salvador	2015/11–2015/12/01	General population- all ages	Outbreak			autochthonous transmission 2015/11
[179]	French Guiana	2015-12-21	General population- all ages				1 case; autochthonous transmission 2015/12
[4,180]	Guatemala	2015/11–2015/12/01	General population- all ages	Outbreak			autochthonous transmission 2015/11
[181,182]	Guadeloupe	1015/11/23–2016/01/21	General population- all ages	Outbreak			1 case confirmed
[169,181]	Guyana	1015/11/23–2016/01/21	General population- all ages	Outbreak			164 cases (45 confirmed) autochthonous transmission 2016/01
[183]	Haiti	2016/01/18	General population- all ages	Outbreak			5 cases, autochthonous transmission 2016/01
[184]	Honduras	2015/12/21	General population- all ages	Outbreak			1 case, autochthonous transmission 2015/12
[179,181]	Martinique	1015/11/23–2016/01/21	General population- all ages	Outbreak			1255 cases (102 confirmed), autochthonous transmission 2015/12
[4,185]	Mexico	2015/11–2015/12/01	General population- all ages	Outbreak			autochthonous transmission 2015/11
[186,187]	Panama	2015/11–2015/12/14	General population- all ages	Outbreak			95 cases, (4 confirmed) autochthonous transmission 2015/12
[4,188]	Paraguay	2015/11–2015/12/01	General population- all ages	Outbreak			6 cases, autochthonous transmission 2015/11 on Brazilian border
[189,190]	Puerto Rico	2015/11/23-2016/01/28	General population- all ages	Outbreak			155 cases (30/73 confirmed), 3 hospitalizations. autochthonous transmission 2015/11
[181]	St. Barthelemy	2015/11/23–2016/01/21	General population- all ages	Outbreak			
[181,182]	St. Martin	2015/11/23–2016/01/21	General population- all ages	Outbreak			1 case confirmed
[4,191, 192]	Suriname	2015/11	General population- all ages	Outbreak			5/6 cases confirmed. autochthonous transmission reported November 2015
[193]	Trinidad	1953–1954: Trinidad	General population- all ages	Prevalence	0.0%	0/15	
[4,194]	Venezuela	2015/11–2015/12/01	General population- all ages	Outbreak			7 cases (4 confirmed) autochthonous transmission 2015/11 on Brazilian border
[195]	Virgin Islands (US)	2016/01/25	General population- all ages	Outbreak			1 case, autochthonous transmission 2016/01
**Travel related- notifiable disease report**							
[196]	New Zealand	2015: New Zealand	General population- all ages	Incidence rate	0.1 per 100,000		6 cases
[197]	New Zealand	2014: New Zealand	General population- all ages	Incidence rate	1.3 per 100,000		57 cases total
[198]	Europe	up to 2016/02/25	General population- all ages			177 cases from 15 countries. Austria (1), Czech Rep (2), Denmark (1), Finland (2), France (66), Germany (20), Ireland (3), Italy (6), Malta (1), Netherlands (30), Portugal (7), Spain (27), Sweden (2), Slovenia (1), UK (8).	

#### Clinical Symptoms, Complications and Pathogenesis

Clinical symptoms associated with ZIKV illness in humans is reported in 72 studies and described in Table 10, including the first experimentally challenged human case recorded in Nigeria (1956) [88] to present cases in South America and the Caribbean region. Thirty four studies report travel related cases in individuals returning from various affected countries. Thirty five studies report on complications with ZIKV, Table 11. Publications that described the clinical features of ZIKV infection in humans were mainly case reports/case series (57/78), epidemiological surveys (12/78), outbreak investigations (11/78), and one human challenge experiment (some studies fell into multiple categories). For most studies (58/78) the total number of cases was <6 while other results summarized the clinical features of up to 13,786 cases. The majority of studies have been published in the past decade and are based on ZIKV acquired infections in South-Central America and Caribbean region (54/75) and Asia/Australasia (48/75) with a number of publications reporting on both regions. A wide range of clinical symptoms are reported for ZIKV infection, most common are mild fever and a maculopapular rash followed by joint pain, headache and more recently bilateral conjunctivitis; see the summary at the bottom of Table 10 for averages across studies. In a recent study, researchers found an association with more severe symptoms in patients that were co-infected with malaria [131]. Six studies reported co-infections with dengue and/or chikungunya [74,116,117,144,146,199], one study reported co-infection with Influenza B and another with Human Immunodeficiency Virus [81,189]. Some of these studies provide evidence for the viremic period, suggested which samples and diagnostic tests to use at various stages of infection and the possible range of incubation periods, however, only one study investigated the immune response in humans [200]. Tappe (2015), investigated the role of cytokines and chemokines in the pathogenesis of ZIKV infected patients from the acute stage (≤ 10 days after symptom onset) to convalescent stage (> 10 days after disease onset) [200].

**Table 10:** Pathogenesis: Studies (n = 72) reporting on clinical symptoms in humans from the first experimentally challenged human in Nigeria (1956) to present (March 1, 2016) cases in the South America and Caribbean region divided by studies with n>6 (25.4%) and n<6 (74.6%).

Ref	Study Year	Country of ZIKV Exposure	N	Fever	Joint pain	Rash Rash	Con-junc-tivitis	Muscle pain	Head-ache	Retro-orbit-al pain	Edema	Lymphadenopathy	Malaise	Asthenia	Sore throat/ cough	Nausea/ vomiting/ diarrhea	Hemato-spermia	COMMENT
**Outbreak Investigations, Cohort or cross-sectional studies**																		
**South- Central America and Caribbean**																		
[173]	2015	Brazil	13786	35%	27%	100%		22%	26%									Summary of the clinical picture from indeterminate acute exanthematous illness in Salvador, Brazil. Many cases not confirmed.
[73]	2015	Brazil	24	38%		86%		54%	58%									
[201]	2015	Brazil	29	45%	38%	72%	79%		17%									14% itchy
[202]	2015	Brazil	10		70%	70%							70%					100% microcephaly in infant.
[76]	2015	Brazil	8	75%	88%	100%		75%	75%	50%	75%	38%						8/8/ Pain scale applied to six cases -pain levels high in most patients, with levels of zero (1/7), seven (2/7), nine (1/7) or 10 (2/7). Duration 2–15 days.
[79]	2014	Easter Island (Chile)	89	100%	100%	100%	100%	100%										Clinical picture of the 89 positive cases on Easter Island (total population 3860)
[177]	2016	Dominican Republic	10	100%	60%	100%	80%	50%	60%				60%					
[188]	2015	Paraguay	6	100%	100%			100%	100%	100%						100%		
[189]	2015–2016	Puerto Rico	30	73%	73%	77%		77%								3%		3% chills and abdominal pain
[61]	2015/10	Suriname	5			100%	100%											
**Asia and Australasia**																		
[144–146]	2013–14	French Polynesia	297	72%	65%	93%	63%	44%	46%	16%	47%			78%	23%	28%		Adenopathies 15%, mouth ulcers 4%
[203]	2014	New Caledonia	6	83%	67%	100%	50%	67%	17%	17%	50%	17%						6/6 itching
[77]	2012	Thailand	7	100%	29%	100%	29%	29%	14%						29%			1/7 Rhinorrhea
[100,147]	2007	Yap Island, Micronesia	31	65%	65%	90%	55%	48%	45%	39%	19%					10%		Clinical picture of 31 confirmed cases identified during a survey of 173 randomly selected households on Yap Island during the outbreak in 2007.
[204]	1977	Indonesia	7	100%	29%		14%	14%				14%	86%			43%		1/7 haematuria, 4/7 anorexia, 2/7 chills, 5/7 stomach ache, 3/7 dizziness, 3/7 constipations, 2/7 hypotension,
**Case Studies < 5 people**																		
**South- Central America and Caribbean**																		
[205]	2016	Brazil	1	100%	100%	100%												bilateral ocular discomfort, blurry vision, and mild redness on day 7
[206]	2015	Brazil	3		33%	33%												Pregnant mothers—no laboratory confirmation
[81]	2015	Brazil	1			100%	100%	100%					100%	100%				Case had HIV.
[207]	2015	Brazil	1	100%		100%	100%				100%			100%				
[52]	2015	Brazil	1	100%		100%		100%										100% itchy
[55]	2015	Brazil	1	100%	100%	100%		100%		100%								100% itchy
[208]	2015	Brazil	1	100%	100%	100%			100%	100%		100%						100% chills
[176]	2015	Colombia	1	100%	100%			100%		100%								100% Abdominal pain and jandice. Comorbidity: Sickle cell disease (>5 yrs). Patient died.
[209]	2015	Colombia	1	100%		100%	100%	100%				100%	100%		100%			
[199]	2015	Colombia	1	100%		100%	100%				100%	100%						Case had co-infection with dengue and chikungunya.
[210]	2016	Costa Rica	1	100%	100%	100%	100%	100%										
[211]	2016	Costa Rica	1	100%	100%	100%	100%		100%									
[210]	2016	Curacao	1			100%	100%	100%										100% diarrhea
[210]	2016	Jamaica	1	100%	100%	100%	100%		100%									100% abdominal pain, retro orbital pain and vomiting
[212]	2016	Martinique, Brazil and Colombia	3	67%	33%	100%	100%	100%						67%	33%	33%		
[210]	2016	Nicaragua	2	100%		100%	100%											
[213]	2015	Suriname	1	100%	100%	100%	100%				100%							100% itchy, painful skin
[214]	2016	Suriname	1	100%		100%					100%		100%	100%		100%		100% itchy and subcutaneous haematomas on arms and legs (day 10) day 29 diagnosed: immune-mediated thrombocytopenia
[215]	2016	Venezuela	2	100%	50%	100%	100%						100%					
[195]	2016	Virgin Islands (US)	1	100%	100%	100%	100%											
**Asia and Australasia**																		
[82]	2010	Cambodia	1	100%					100%						100%			
[216]	2015	Cook Islands	2	100%		100%	50%	100%	100%									
[57]	2014	Cook Islands	1		100%	100%							100%	100%		100%		
[217]	2014	Cook Islands	2	100%	100%	100%			100%					100%				
[218]	2014	French Polynesia	2	100%	100%	100%	100%	100%			100%	100%	100%	100%				1/2 slight gingival bleeding for a few days
[74]	2014	French Polynesia	2	100%	100%	50%	50%	100%	50%	50%				100%		50%		2 cases had co-infection with two different dengue strains.
[219]	2014	French Polynesia	1		100%	100%	100%						100%					
[59]	2013	French Polynesia	1	100%	100%	100%		100%	100%									
[220]	2013	French Polynesia	1	100%	100%	100%	100%	100%						100%				
[221]	2013	French Polynesia	1	100%	100%				100%					100%			100%	
[72]	2013	French Polynesia	3	100%	100%	100%	67%		100%		33%			100%				1/3 aphthous ulcer
[222]	2013	French Polynesia	2	50%		100%		50%										Pregnant cases had ZIKV infection immediately before or after birth; newborns developed ZIKV (2/2 RT-PCR positive, 1/2 rash) with no complications (one had gestational diabetes)
[223]	2013	French Polynesia	1	100%		100%	100%	100%										Developed Guillain-Barré syndrome at day 7 (see complications table)
[69]	2013	French Polynesia	2	100%	50%	100%	50%		50%	50%								
[67]	2013	French Polynesia	1	100%	100%	100%	100%	100%				100%						
[224]	2013	French Polynesia	1	100%	100%	100%	100%						100%	100%				
[53]	2014–15	Indonesia	1	100%	100%			100%	100%				100%					
[78]	2015	Indonesia	1	100%		100%	100%	100%										
[66]	2012	Indonesia	1			100%	100%	100%	100%						100%	100%		
[225]	2014	Malaysia	1	100%	100%	100%	100%				100%				100%			1/1 burning sensation of palms of hands and soles of feet. 1/1 sudden development of bilateral dull and metallic hearing—left ear a very short delay between a sound and perception of sound, duration 10 days with gradual resolution.
[58]	2015	Maldives	1	100%	100%	100%				100%								
[226]	2015	New Caledonia	1	100%		100%												Gave birth, 37 wk, at ZIKV onset.
[75]	2012	Philippines	1	100%			100%	100%	100%					100%	100%			1/1 stomach pain, anorexia
[227]	2014	Thailand	2	50%		100%	50%		100%	50%	50%							50% diffuse pain
[228]	2016	Thailand	1	100%					100%									
[229]	2014	Thailand	1	100%		100%	100%		100%									
[60]	2013	Thailand	1	100%	100%	100%	100%	100%	100%									1/1 chills, 1/1 mouth blisters (appeared after 2 days); symptomatic for 16 days
[230]	2013	Thailand	1	100%	100%	100%					100%			100%				
**Africa **																
[127]	1971	Nigeria	2	100%	50%				50%									
[88]	1956	Nigeria	1	100%					100%				100%					Laboratory challenge of human volunteer
[43]	1952	Nigeria	3	100%	67%				67%	33%					33%			1/3 jaundice (but concurrent epidemic of jaundice), 1/3 albumin in the urine on day 3–5.
[231]	2008	Senegal	3		100%	100%	33%	67%	100%		66%		33%	66%			50%	2/3 chills, 1/3 prostatitis, 2/3 aphthous ulcers on lip (day 4), 1/3 photophobia, 2/3 reported arthralgia reoccurring for several months.
[232]	1962	Uganda	1	100%	100%	100%		100%	100%				100%					
**Europe**																	
[233]	1973	Portugal	1	100%	100%					100%								Laboratory acquired ZIKV: 1/1/ chills, 1/1 sweating
**Symptom Frequency across case studies**				**84%**	**57%**	**79%**	**54%**	**43%**	**41%**	**13%**	**16%**	**10%**	**22%**	**28%**	**11%**	**7%**	**3%**	
**Symptom Frequency across Observational studies**				**66%**	**54%**	**85%**	**41%**	**45%**	**31%**	**15%**	**13%**	**5%**	**14%**	**5%**	**3%**	**12%**	**1%**	

**Fever** (typically reported as low grade 38–40 C lasting usually 2–4 days, range 1–8 days).

**Joint pain** (usually wrist, fingers, ankles or knees affected, some reports of low back pain. Pain lasts typically less than a week, but there are reports of intermittent arthralgia for 1–2 months).

**Rash** (common description: diffuse pink maculopapular rash which covered the face, neck, trunk and upper arms, occasionally this is reported as "itchy", typically appeared around day 2 of illness and faded around day 5, although there are cases where the rash lasted up to 2 weeks.).

**Conjunctivitis** (red eyes)—mostly reported to occur in both eyes.

**Muscle pain** (reported to range from mild to severe and last 2–7 days).

**Headache** (mild headache often the first symptom and is reported to last 2–4 days).

**Retro-orbital pain**. Edema (usually in hands/fingers or ankles/feet) duration approximately 7 days.).

**Lymphadenopathy**—duration up to 2 weeks. Often reported in cervical area.

**Malaise** 2–14 days.

**Asthenia**—2 to 14 days and often preceded rash by up to 48 hours.

**Sore throat/cough** (duration 4 days).

**Nausea/vomiting/ diarrhea**.

**Hematospermia** (occurred at day 5 to 14 after symptoms appeared)

**Table 11:** Complications reported to be associated with ZIKV infection in 35 case reports/ case series and outbreak reports. 20 reports on birth defects and microcephaly in pregnant women (2013–2016) and 24 on Guillain-Barré syndrome following ZIKV infection (probable and confirmed) during outbreaks in the Pacific Islands and the Americas (2011–2016).

Ref	Country	Study year	Complication	N	ZIKV confirmed?	Description of clinical findings:
**Microcephaly and other birth defects potentially associated with ZIKV infection**						
[148]	French Polynesia	2014	Birth Defects: fetal cerebral anomaly	18	4/6 amniotic fluid samples were RT-PCR positive	French Polynesia has ~4000 births/year. Retrospective analysis of birth defects involving the central nervous system indicated 18 cases in 2014 (vs. 4 in 2013 and 3 in 2012) where the mothers may have been infected with ZIKV early in pregnancy. Amniotic fluid samples from standard testing procedures showed 4/6 samples were ZIKV positive. Since our search, an additional paper on this set of cases has been published.
[206]	Brazil	2015	Birth defects: microcephaly	3	33% mothers had clinical symptoms. No testing done.	CT scan and ocular examination showed all infants had unilateral ocular findings: gross macular pigment mottling and foveal reflex loss. Well-defined macular neuroretinal atrophy was detected in one child.
[80]	Brazil	2015	Birth defects: microcephaly	8	25% positive amniotic fluid, 75% mothers had clinical symptoms during pregnancy	Amniotic fluid positive fetuses (n = 2), note mother’s serum RT-PCR^1^ was negative: both eyes had cataracts and intraocular calcifications, and one eye was smaller than the other. Fetuses from mothers with clinical symptoms (n = 6): fetal neurosonograms showed 33% cases with cerebellar involvement and 16% with severe arthrogryposis.
[201]	Brazil	2015	Birth defects: Microcephaly and ocular defects	29	29 cases	29 infants age 1–6 months. 23/29 mothers had clinical ZIKV infections: 18/29 first trimester, 4/29 second trimester, 1/29 third trimester. 6 had no symptoms of ZIKV; 10 patients had ocular findings and were presumed to have been exposed to ZIKV.
[52]	Brazil	2015	Birth defects: Microcephaly	2	2 cases: first and second trimester ZIKV infections	Case 1: normal ultrasound at 16wk, ZIKV at 18wk, ultrasound at 21 weeks detected microcephaly, confirmed at 27wk. Baby born at 40wks with head circumference of 30cm. Case 2: ZIKV at 10wk, 22wk ultrasound indicated fetal head <10th percentile, 25wk indicated microcephaly, term delivery neonate presented with severe ventriculomegaly, microphthalmia, cataract, and severe arthrogryposis in the legs and arms. Amniotic fluid positive at 28wks.
[55]	Brazil	2015/10	Microcephaly	1	RT-PCR positive on fetal brain sample	Mother had ZIKV at 13 weeks gestation. Ultrasound at 14 and 20 weeks were normal. Ultrasound at 29 and 32 weeks showed retardation of growth with normal amniotic fluid and placenta, a head circumference below the second percentile for gestation (microcephaly), moderate ventriculomegaly. Brain structures were blurred, calcifications and no other fetal structural abnormalities. Fetal, umbilical, and uterine blood flows were normal. RT-PCR^1^ ZIKV positive in the fetal brain sample (6.5×107 viral RNA copies per milligram of tissue).
[202]	Brazil	2015/12	Birth defects: Microcephaly and ocular findings	10	10 cases	10 cases had clinical diagnosis of ZIKV vertical infection (mothers 7/10 rash, 6 in 1st trimester) and diagnosed with ophthalmological abnormalities
[234]	Brazil	2015/08–2015/10	Birth defects: Microcephaly	35	35 cases. 25/35 severe microcephaly, 17/35 at least one neurologic abnormality.	26/35 mothers recalled a rash during pregnancy: first trimester 21/26 and 5/26 in the second trimester. Pathology: Computed tomography scans and transfontanellar cranial ultrasounds showed a consistent pattern of widespread brain calcifications, ventricular enlargement secondary to cortical/subcortical atrophy, excessive and redundant scalp skin in 11 (31%) cases, also suggests acute intrauterine brain injury, indicating an arrest in cerebral growth.
[235]	Brazil	up to 2015/11/21	Birth defects: Microcephaly	739	739 cases (1 death)	Nov 7, 2015: case definition revised from <33cm to <32cm.
[4,172]	Brazil	up to 2015/11/30	Birth defects: Microcephaly	1248	1,248 cases (7 deaths)	1,248 cases equates to 99.7/100,000 live births have microcephaly. Brazil noted many affected women appear to have been infected with ZIKV in first trimester (no data)
[236]	Brazil	up to 2015/12/05	Birth defects: Microcephaly	1761	1761 cases (19 deaths)	
[170, 237]	Brazil	up to 2016/01/31	Birth defects: Microcephaly or CNS malformation	4783	4783 cases (76 deaths)	5/76 deaths were ZIKV positive. Historic average 163/year.
[116,117,238]	Brazil	2015/11–2016/02/13	Birth defects: Microcephaly or other CNS involvement	5280	5280 cases (108 deaths)	Brazil 2001 to 2014 had an average of 163 microcephaly cases/year. Validation of 1345 cases of microcephaly is complete: 837 discarded, 508 confirmed by 421/462 cases radiological findings and 41/462 ZIKV confirmed infection.
[48]	Brazil	2016	Birth defects: Microcephaly	1	1 case	20 year old mother: microcephaly detection at 18 weeks, pregnancy terminated at 32 weeks. Fetal tissues: cerebral cortex, medulla oblongata and cerebrospinal and amniotic fluid ZIKV RT-PCR positive.
[116, 239]	Hawaii	2016/01/08	Birth defects: Microcephaly	1	Case of microcephaly, confirmed ZIKV	The mother likely acquired ZIKV in Brazil (May 2015) and her newborn acquired the infection in utero.
[240]	USA	2015/08/01–2016/02/07	Birth defects: Microcephaly, miscarriage	9	3/9 fetuses ZIKV positive.9/9 mothers ZIKV confirmed.	1st trimester (6/9): 2 miscarriages, 2 terminations, 1 microcephaly case, one not born yet. Trimester 3 (3/9): 3 apparently healthy infants. Travel to American Samoa, Brazil, El Salvador, Guatemala, Haiti, Honduras, Mexico, Puerto Rico. In USA 151/257 pregnancies tested for ZIKV, 8 IgM positive.
**Guillain-Barré syndrome (GBS) or other neurological complications potentially associated with ZIKV infection**						
[116, 117, 170, 172,241,242]	Brazil	2015/11	GBS	7	7/10 GBS patients were ZIKV positive	1708 GBS cases (2015) vs. 1439 cases (2014).
[4,116, 117,170,172,237,242]	Brazil	2015/05/01–2015/07/13	GBS	42	62% (26/42) were ZIKV positive	
[173]	Brazil	2015/02/15-2015/06/25	GBS	24		24 cases of suspect GBS were reported during an outbreak of 14,835 cases of “indeterminate acute exanthematous illness” in Salvador, Brazil (3rd largest city). Confirmation tests not done for ZIKV, chikungunya or dengue.
[116,117, 175,237]	Colombia	2015/12/27–2016/01/31	GBS	86	86 cases in 2 months	Historic average is 242 GBS /year (20 cases /month).
[117,178,237,242, 243]	El Salvador	2015/12–2016/01/09	GBS	46	46–118 GBS cases in 5 weeks (2 deaths)	2014 average was 169 cases/year. Case series 12/22 GBS patients had ZIKV within the 15 days prior to GBS.
[116,117,137,138, 144–147, 172,242,244,245]	French Polynesia	Nov 2013-Feb 2014	GBS	42		42 cases of GBS and increased incidence of neurological complications were reported associated with the ZIKV outbreak in the Pacific Islands. 37/42 GBS cases reported having a viral syndrome 6 (4–10) days before the onset of GBS. GBS symptoms peaked at 6 (4–9) days and by 3 months after discharge, 24 (57%) patients were able to walk without assistance. All GBS patients were hospitalized, median 11days (7–20) (N = 42) and 51 days (16–70) for ICU patients (n = 10).Symptoms: Clinical presentation at hospital admission included generalised muscle weakness (74%), inability to walk (44%), facial palsy (64%), 39 (93%) patients had increased (>0·52 g/L) protein concentration in their CSF, 16 (38%) patients were admitted to ICU and 12 (29%) required respiratory assistance. All cases (100%) received immunoglobulin treatment and one (2%) had plasmapheresis.
[245]	French Polynesia	Nov 2013-Feb 2014	GBS	42	Case control study:	If the ZIKV attack rate in French Polynesia was 66%, the risk of GBS was 0·24/1000 Zika virus infections. Patient and control samples drawn at several time points were examined by RT-PCR, IgM / IgG and PRNT^2^ reaction. 98% of GBS patients were IgM or IgG positive, 19% cross-reacted with dengue, but 100% were confirmed ZIKV positive by PRNT. Compared to a control group of hospitalized patients, the odds of a GBS patient being ZIKV positive was 59·7 (95% CI 10·4 –+∞); p<0·0001. And for PRNT the odds was 34·1 (95% CI 5·8 –+∞) p<0·0001. No association was detected for dengue test results and GBS.
[223]	French Polynesia	2013	GBS	1	Case report, sero-positive	Polynesian woman, early 40s had ZIKV symptoms 7 days before neurological symptoms. No past medical history except acute articular rheumatism. Day 0: evaluated for paraesthesia of the four limb extremities. Day 1: admitted to hospital, paraesthesia had evolved into ascendant muscular weakness suggestive of GBS. Day 3: developed tetraparesis predominant in the lower limbs, with paraesthesia of the extremities, diffuse myalgia, and a bilateral, but asymmetric peripheral facial palsy. Deep tendon reflexes absent. No respiratory or deglutition disorders. Chest pain developed related to a sustained ventricular tachycardia, and orthostatic hypotension, both suggestive of dysautonomia. Electromyogram confirmed a diffuse demyelinating disorder, with elevated distal motor latency, elongated F-wave, conduction block and acute denervation, without axonal abnormalities. Day 13: discharged with paraparesis requiring the use of a walking frame, and the facial palsy slowly disappeared. Day 40, able to walk without help and muscular strength score was 85/100.
[181, 237]	Martinique	2016/01/21	GBS	6	6/6 GBS cases were ZIKV positive	
[246]	New Caledonia	2011/01/01–2014/12/31	GBS	42	42 cases of GBS between 2011–2014 investigated.	42 cases of GBS: incidence 2011 = 2.6 (0.66–4.54)/100000 vs. 2014 = 5.09 (2/49-7.56)/100000 = NOT a significant difference. 13 (30%) cases occurred between March and July 2014 (during ZIKV outbreak), 6 in April 2014. These patients indicated 2 confirmed and 2 suspect ZIKV cases and 4 dengue cases preceded GBS.
[189]	Puerto Rico	2015/11/23-2016/01/28	GBS	1	1 ZIKV positive case developed GBS	
[116, 117,170]	Suriname	2015/12–2016/01/21	GBS	13	10 GBS (2015) and 3 GBS (2016)	Historic average is 4/year. 2/10 2015 cases were ZIKV confirmed.
[214]	Suriname	2016/01	immune-mediated thrombocytopenia	1	ZIKV positive patient	Developed normal clinical symptoms of ZIKV, and on day 29 diagnosed immune-mediated thrombocytopenia.
[117, 170,175,237]	Venezuela	2016/01/01–2016/02/10	GBS and other neurological symptoms	252	252 GBS (3 confirmed)	Up to 76% of patients reported clinical symptoms of ZIKV. 65% had comorbidities. 3 cases with other neurological symptoms were ZIKV positive.

^1^ RT-PCR = reverse transcription-polymerase chain reaction^,^

^2^ PRNT = plaque reduction neutralization test

Complications associated with ZIKV infection in humans are reported in Table 11. The research and attention concentrated on all potential complications of ZIKV has increased significantly in the last few months; we identified that many official reports and papers published the same information multiple times. A number of small case series or case studies investigating complications with ZIKV have demonstrated a chronological association between ZIKV infection in pregnant women and development of microcephaly in the fetus, ZIKV infection in fetuses and newborns from mothers exposed to ZIKV at various points during pregnancy, and ZIKV preceding neurological conditions, mainly Guillain-Barre syndrome in adults, Table 11. A significant increase in microcephaly has been reported in Brazil and a good deal of research and scrutiny of the existing data is on-going to determine the degree that ZIKV is contributing to microcephaly cases. Two studies discuss the criteria used to categorize infants as positive for microcephaly and the potential impact on the numbers reported in Brazil [247,248]; one study shows evidence of an increase in microcephaly in Brazil starting in 2012 prior to ZIKV arriving in the Americas [248]. Other birth defects have also been noted including a number of ocular abnormalities and hydrops fetalis [48,201]. Hopefully on-going cohort studies will provide better evidence for the role ZIKV plays in birth defects and fetal death. Outside of Brazil, no other country has noted the increase in microcephaly at the time of writing (March 1, 2016), however several countries in the Americas and Australasia have noted an increase in Guillain-Barré Syndrome coinciding with a ZIKV outbreak, Table 11 [146,242,246].

#### Transmission

ZIKV is known to be transmitted by mosquitos; however there is also increasing evidence that intrauterine transmission and sexual transmission via semen may occur, Table 12. It has also been suggested that transmission by blood transfusion is possible, although no cases have been reported [71]. Two studies examined the efficacy of amotosalen combined with UVA light treatment of plasma and recommended that it was effective to deactivate ZIKV [30,32].

**Table 12:** Studies reporting various modes of human to human transmission and one human to mosquito transmission study.

Refid	Year of study	Country	Study design	Mode of transmission	Comment
[80]	2015	Brazil	Case study	human- intrauterine	2 mothers with clinical symptoms of ZIKV were serum negative, but amniotic fluid positive.
[116]	2016	Brazil	Outbreak investigation	human- intrauterine	17 infants with microcephaly and 5 fetuses were ZIKV positive
[52]	2016	Brazil	Case study	human- intrauterine	Mothers presented with ZIKV at 10 and 18 weeks. Fetuses developed microcephaly after ZIKV infection and were positive for ZIKV in fetal tissue
[226]	2015/07	New Caledonia	Case study	human- breast milk	No transmission was noted, but virus was cultured from breast milk.
[71]	2013/11/21-2014/02/17	French Polynesia	Cross-sectional	human—blood transfusion	42/1505 blood donations were ZIKV positive, 11/42 donors reported having ZIKV like symptoms within 10 days of the donation.
[222,242]	2013/12–2014/02	French Polynesia	Case study	human- intrauterine	2 mothers had active ZIKV infections at delivery. Transmission was thought to have occurred either intrauterine or during delivery.
				human- direct contact	
				human- breast milk	
[103]	1979/10–1980/03	Gabon	Prevalence	human- intrauterine	1/28 mother-newborn sera pairs positive for ZIKV.
[215]	2016	United States	Case study	human—sexual transmission	7 days after first patient developed symptoms, patient 2 developed symptoms.
[227]	2016	Italy	Case study	human—sexual transmission	19 days after patient one developed symptoms, patient 2 developed symptoms.
[231]	2008	United States	Case study	human—sexual transmission	Hematospermia reported in case 1 and is thought to have transmitted ZIKV to case 3 by sexual transmission. Another possibility is that direct contact and exchange of other bodily fluids, such as saliva, could have resulted in ZIKV transmission, but illness did not develop in their 4 children.
[88]	1956	Nigeria	Challenge trial	human- mosquito	Single experimental case was unsuccessful at transmission of ZIKV to *Ae*. *aegypti*, possibly because the case had a very low titre.

#### Testing for ZIKV infection

Serum samples were most commonly used to diagnose ZIKV infection, but other samples including saliva, nasopharyngeal swabs, urine, semen, amniotic fluid and breast milk have also been used to detect ZIKV infection with RT-PCR, Table 13. There were very few studies (9) that evaluated a diagnostic test for ZIKV and of those only 33% reported data on test performance and none reported on a commercial test, Table 14. Of note, several studies captured in this review discuss potential cross-reactivity of some dengue serology tests during the acute phases of ZIKV infection, therefore laboratories and physicians need to follow readily available guidelines [249] so appropriate samples are taken for accurate diagnosis [208,211,212,227].

**Table 13:** Non-serum samples for ZIKV rtPCR testing reported in 29 studies 2013–2015.

Ref	Year of study	Country	Sample (N)	Test	Days since symptoms appeared	Comment
[78]	2015	Australia	Nasopharyngeal	RT-PCR^1^	2	
[80]	2015	Brazil	Amniotic Fluid	RT-PCR	NR	2 mothers with clinical symptoms of ZIKV were serum negative, but amniotic fluid positive.
[238]	2016	Brazil	Urine	RT-PCR	NR	ZIKV detected in urine and saliva samples. Transmission potential unknown
[52]	2015	Brazil	Urine	RT-PCR	70–126	Negative Urine
			Amniotic Fluid	RT-PCR	70–126	Positive amniotic fluid
[49]	2016	Brazil	Urine	RT-PCR	4–14	Urine positive 4–14 days (7 days longer than serum)
[4]	2015	Brazil	Fetal organs	RT-PCR	NR	Positive: blood and organ samples (brain, liver, spleen, kidney, lung, and heart).
[56]	2015	Brazil	Fetal brain and placenta	RT-PCR	NR	Only fetal brain tissue was positive
[168,242]	2016	Brazil	Fetal tissue	RT-PCR	NR	Tissue samples were positive
[172]	2015	Brazil	Fetal tissue	RT-PCR	NR	Only central nervous system samples were positive. Organs (heart, lung, liver or placenta) were negative.
			Amniotic Fluid	RT-PCR	NR	Amniotic fluid was positive
[60]	2013	Canada	Urine	RT-PCR	6	Patient was RT-PCR serum positive on day 6 and 9, IgM positive from day 77 onward.
			Nasopharyngeal	RT-PCR	6	
[143]	2015	Fiji	Saliva	RT-PCR	NR	ZIKV positive, sample taken for diagnosis
[58]	2015	Finland	Urine	RT-PCR	7	RT-PCR of serum was negative
[212]	2015–2016	France	Urine	RT-PCR	3	Blood, urine and saliva positive on day 3 and sero-conversion occurred day 8.
			Saliva	RT-PCR	3	
[145,146,220,250]	2013–2014	French Polynesia	Saliva	RT-PCR	3 (1–8)	19.2% tested positive by saliva while negative in blood; 8.8% tested positive in blood while negative by saliva (n = 319), McNemar test, p = 0.0117. Oral swabs are non-invasive; sensitivity of testing is increased by collecting both oral swabs and blood samples.
[222]	2013–2014	French Polynesia	Breast Milk (2)	RT-PCR	3–8	Breast milk in two cases positive on day 3–8. Sequential samples not done.
** **			Urine	RT-PCR	8	Mother and infant urine positive on day 8, negative day11
** **			Saliva	RT-PCR	3	Infant and mother positive on day 3 of illness.
[221]	2013	French Polynesia	Urine	RT-PCR	17	RT-PCR of serum negative
			Semen	RT-PCR	14–17	
[148]	2013/09-2014/03	French Polynesia	Amniotic Fluid	RT-PCR	NR	Retrospective evaluation of amniotic fluid samples from cases of fetal cerebral dysfunction
[229]	2014	Japan	Urine	RT-PCR	7	RT-PCR of serum negative
[69]	2013–2014	Japan	Urine	RT-PCR	NR	RT-PCR of serum negative, first time ZIKV virus particles reported in urine.
[214]	2016	Netherlands	Urine	RT-PCR	17–18	Urine positive and serum negative
[203]	2014	New Caledonia	Urine (6)	RT-PCR	10 to >20	Urine estimated viral load: 0.7–220.10^6^ copies/ml. Detectable in urine about 7 days longer than serum. Infectious particles not isolated.
[226]	2015/07	New Caledonia	Breast Milk	RT-PCR	4	ZIKV RT-PCR positive in serum on day 3, in breast milk on day 4 and was confirmed by the presence of a cytopathic effect and by RT-qPCR 39 million RNA copies per mL
[116,237,251]	2016	Martinique	Urine	RT-PCR	NR	Urine sample part of routine sample collection.
[55,238]	2015	Slovenia	Fetal organs and tissue	RT-PCR	133	Only fetal brain tissue positive. Tested: placenta, lungs, heart, skin, spleen, thymus, liver, kidneys, and cerebral cortex.
[217]	2014	United Kingdom	Semen	RT-PCR	28	May be a good sample if the initial viremic blood samples are not available.
[239]	2016	United States	Cerebrospinal fluid	IgM & PRNT	NR	Mother and fetus were IgM and PRNT^2^ positive.

^1^ RT-PCR = reverse transcription-polymerase chain reaction

^2^ PRNT = plaque reduction neutralization test

**Table 14:** Nine studies evaluated the performance of diagnostic tests for ZIKV—none were reported to be commercially available and in most studies evaluation was not on clinical samples.

Ref	Country	Year published	Diagnostic tests compared/evaluated	Data available?	Comment
[212]	France	2011-Present	Dengue false positive	insufficient data	Protocol for ZIKV testing given dengue IgM false positive during acute ZIKV infection.
[96]	Senegal	2011-Present	RT-PCR^1^: one step / quantitative	Yes: Sn^4^, raw data, detection limits	Developed a rapid, sensitive and specific real time PCR for the detection of ZIKV circulating in Africa and Asia
[252]	Singapore	2011-Present	RT-PCR	Yes: Sp^4^, detection limits	Laboratory Sn/Sp good, but not tested on clinical samples
[253]	Senegal	2001–2010	RT-PCR: one step	Yes: Sn, detection limits	Lab evaluation only, not tested on clinical isolates.
[62]	Yap Island, Micronesia	2001–2010	Clinical diagnosis (by signs and symptoms), serological tests (IgM and IgG), PRNT^2^, RT-PCR	insufficient data	Describes that IgM cross-reactivity with dengue occurs when ZIKV infection occurs in individuals seropositive for dengue. (This has since been reported in a number of case studies where dengue IgM initially climbs, but then decreases and the patient never sero converts to IgG dengue, but will develop IgG to ZIKV)
[24]	France	1991–2000	Sequencing the virus, RT-PCR	insufficient data	Single set of primers for universal amplification of Flaviviruses: based on conserved elements in the 3' untranslated region of mosquito-borne Flavivirus (YF, DEN, JE, WN and ZIKV)^3^ RNA and in the protein NS5. Recombinant plasmids can then be used to identify the specific virus in one round of rtPCR with hybridization in 2 days.
[104]	Senegal	1981–1990	Serological tests (IgM), complement fixation test	Yes: Sp	IgM combined with virus isolation is recommended for surveillance as specific diagnosis from a single serum sample is possible.
[254]	England	1971–1980	Cross-neutralization	insufficient data	Older study, technique obsolete
[255]	England	1960–1970	Micro-culture method	insufficient data	Older study, technique obsolete

^1^ RT-PCR = reverse transcription-polymerase chain reaction

^2^ PRNT = plaque reduction neutralization test

^3^ YF = yellow fever; DEN = dengue fever; JE = Japanese encephalitis; WN = West Nile virus

^4^ Sn = sensitivity, Sp = specificity

From the studies (n = 76) where details of cases and diagnosis were reported, RT-PCR was most frequently used (68.4%) followed by serology (40.8%) and virus isolation (3.9%); one study conducted a serum cytokine analysis [200]. Among the studies that used serology (n = 31), IgM was used in 80.6% of studies, IgG in 41.9% and plaque reduction neutralization tests (PRNT) in 67.7%. Four studies published before 1982 used hemagglutination-inhibition tests, complement fixation tests and neutralization tests [127,204,232,233].

## Discussion

In this scoping study we identified 233 primary research papers, reports, theses and conference proceedings on ZIKV published between 1952 and March 1, 2016. There is evidence that the Asian lineage of ZIKV, which had been historically shown to be circulating in Malaysia, Philippines, Pakistan, Thailand and Cambodia, caused an outbreak on Yap Island in 2007 and continued to spread, affecting a number of the Polynesian islands from 2012 to present. In 2014 the virus traveled to South America where autochthonous circulation was recognised in Brazil in early 2015. At this time (March 2016), 31 countries in South-Central America and the Caribbean are reporting local transmission [3].

Historically ZIKV was not known to cause severe illness in humans and for the first 60 years since its initial identification in Uganda (1947) there were only a few accounts of illness caused by ZIKV. These were mainly in laboratory workers who inadvertently became exposed to the virus. Epidemiological studies in this time period were mainly conducted in Africa and evaluated populations for seropositivity to ZIKV, but not active infection. In most countries where the population was tested for ZIKV, researchers found a proportion of the population had been exposed. This proportion varied widely and was likely affected by the types of samples and tests used, a variety of climatic factors, vectors present and their abundance, and other competing viruses circulating in the area. ZIKV can circulate at the same time as other viruses e.g. dengue and Chikungunya, sharing the same vector, however there is no evidence that exposure, infection or immunity to one virus impacts the outcome of a ZIKV infection or provides protection.

During the recent outbreaks from 2007 to present, reporting of data concerning clinical characteristics of ZIKV has significantly increased. Based on the Yap Island outbreak, 18% of exposed individuals are likely to experience symptoms, thus there is likely substantial under-reporting of ZIKV infections as 80% of infected individuals may be asymptomatic [100]. Symptoms are usually mild and very similar to other co-circulating viruses (dengue and chikungunya), which makes diagnosis based on clinical symptoms challenging [173]. The World Health Organization released its interim case definition for ZIKV as a person presenting with clinical symptoms: rash or fever and one or more of arthralgia, arthritis or conjunctivitis and a positive IgM with epidemiological link (probable case) or a sample positive for ZIKV RNA or IgM with a positive PRNT for ZIKV vs. other flaviviruses [256]. However clinical identification of ZIKV is difficult due to similarities with other co-circulating viruses (e.g. dengue and chikungunya) and testing is currently the responsibility of specialized laboratories [257].

Potential complications reported following ZIKV infection are of great concern to the public health community. Associations with birth defects and neurological complications have been reported relatively recently causing the World Health Organization to issue an alert for precautionary measures and intense investigation to close the knowledge gaps associated with ZIKV potential to cause birth defects; mainly microcephaly [4,258–260]. Reports of increased incidence of GBS and other neurological complications were noted in the French Polynesian and New Caledonia outbreaks [137,223,246] and have since been noted in several countries in South and Central America [117,173]. Currently Brazil, El Salvador, Venezuela, Colombia and Suriname have reported an increased incidence and Puerto Rico and Martinique have reported ZIKV associated cases of GBS [117,237,261]. A number of studies are underway to try to understand who is at high risk of developing GBS and other neurological symptoms following ZIKV infection [245]. Although birth defects such as microcephaly linked to ZIKV have been reported in Brazil and possibly from French Polynesia [148], it is not known why microcephaly has not been reported by other affected countries. This may be a matter of population size, a lag time between ZIKV spread and the birth of affected infants, and a lack of awareness that ZIKV may impact fetal development. Hopefully the retrospective and prospective studies underway in Brazil and other ZIKV affected countries will improve our understanding of the role of ZIKV in causing microcephaly, other birth defects and negative pregnancy outcomes as this is a current knowledge gap.

Guidelines have been developed for the diagnosis of ZIKV infection in humans and based on the studies captured in this review RT-PCR is most common on serum samples and is recommended for samples collected within the first 7 days of symptoms. Many studies were identified that used other samples (saliva, nasopharyngeal swabs, urine, cerebral spinal fluid, semen, amniotic fluid and breast milk) and RT-PCR to identify ZIKV RNA which would be indicative of ZIKV infection. Studies on some of these other sample types may offer a longer or later window of sampling for identification of the virus RNA with RT-PCR, but none have been widely used within primary research studies to date. The identification of ZIKV RNA in various types of bodily fluids raises questions of human to human transmission via saliva, semen and breast milk. At this time only sexual transmission via semen has been reported. ZIKV has been identified in semen up to 72 days post symptoms; however, this is based on a small number of observations and further research is needed to confirm and better understand this finding. Given the potential for ZIKV survival in semen for a long period of time, current guidelines suggest precautions are taken for up to 6 months following possible exposure to ZIKV to try to prevent some of the growing number of sexually transmitted cases reported from Europe and North America [215,221,227,262–264]. Intrauterine transmission has also been shown to be highly plausible. The first case study was from French Polynesia in women who were already infected at time of delivery and infection was confirmed in both mothers and newborns; the newborns recovered from ZIKV infection without complications [222]. Studies from Brazil and other countries have now provided further evidence for the impact of ZIKV on fetuses in utero as several studies have reported ZIKV in amniotic fluid and deceased fetuses and infants with severe birth defects [55,80,172,214,222,240,251]. The implications of ZIKV infection during gestation and variability in the severity of birth defects depending on when maternal ZIKV exposure occurs during pregnancy are current knowledge gaps that are the focus of research progressing in the Americas [48,52,55,234,240,259]. Based on the first few case series of women exposed to ZIKV during pregnancy with documented fetal/infant outcomes, the data suggest ZIKV infection up to 20 weeks of gestation may be the highest risk for negative outcomes such as fetal death or birth defects [48,52,55,234,240,259]. Transmission by blood transfusion is also possible, although there are currently no documented cases of this type of transmission. There are studies from French Polynesia indicating the possible risk of transfusion due to subclinical viremic blood donors. Two studies demonstrated the efficacy of photochemical treatment of plasma to prevent plasma transfusion-transmitted ZIKV and potentially other arboviruses [30,32,71].

Only 56 publications on ZIKV preceded the 2007 Yap Island outbreak, which was the first time that wide spread illness due to ZIKV was documented. This research was primarily published in Africa and focused on vectors, hosts, and understanding the virus rather than on the burden of illness in humans. These articles provide evidence for the vector competence of several mosquito species to be infected with ZIKV and their ability to transmit ZIKV. They also suggest that non-human primates can be infected with ZIKV and may have a role in its transmission cycle. However, there is not enough evidence to conclude whether non-human primates are a reservoir or incidental host of ZIKV [23]. Very few surveys have been conducted on other animal species and the literature doesn’t suggest other potential reservoirs including bats. Thus, identification of the reservoirs or potential spectrum of hosts for ZIKV remains a knowledge gap.

Molecular evaluation of ZIKV through time has been carried out in a few studies and suggest that ZIKV emerged in Uganda around the 1920s and demonstrated little preference for host or vector during its spread through west and central Africa and Asia [68,83]. They demonstrated that the ZIKV cycle may be 1–2 years compared to 5–8 year cycles seen by other viruses [68]. To date a significant change in the ZIKV genome that lead to increased pathogenesis in humans, increased virulence or spread to new competent vectors has not been described in the literature. Although given the range of vectors shown to be competent for ZIKV transmission, it is possible that ZIKV recently started cycling in a vector species it historically had not. Further research on the evolution of ZIKV and competent vectors may explain this knowledge gap.

Mitigation strategies to prevent or control ZIKV were the topic of one experimental study examining the complexity of a resistance gene in *Ae*. *aegypti formosus* and concluded that the resistance to ZIKV in this mosquito was complex and not easily transferred to other mosquito species [31]. Although general mosquito mitigation is outside the scope of this review, it is relevant to the prevention of ZIKV and other mosquito transmitted diseases.

This scoping review identified and characterized the global literature on ZIKV (up to March 1^st^, 2016) and identified several knowledge gaps with respect to its epidemiology, the burden of disease in humans and complications related to ZIKV infection. Historically there has been little research on this virus, however, given the current spread of ZIKV through Australasia and the Americas, significant research resources have been allocated to closing many of the knowledge gaps identified in this scoping review. Future updates of this review will likely demonstrate enhanced evidence and understanding of ZIKV and its impact on public health.

## Supporting Information

S1 File Protocol for the Scoping Study of Zika Virus literature (DOCX)

S2 File Dataset for the Scoping Study of Zika Virus literature. (XLSX)
