# Supplementary material for: Scoping Review of the Zika Virus Literature
Source: PLoS One. 2016 May 31;11(5):e0156376. doi: 10.1371/journal.pone.0156376 (PMC4887023; doi:10.1371/journal.pone.0156376)
Supplement: S1 File — (DOCX) [file pone.0156376.s001.docx]

# Title:

*What is the current state of the evidence on Zika virus pathogenesis, epidemiology, risk factors, diagnosis, surveillance methods, prevention and control strategies and, knowledge, societal attitudes and perceptions towards infections in humans, mosquito vectors and animal reservoirs?*

# Authors:

Lisa Waddell^1^, Judy Greig^1^

^1^Public Health Risk Sciences Division of the Laboratory for Foodborne Zoonosis, Public Health Agency of Canada.

Contact: Lisa Waddell, Tel:226-979-7174 or email: lisa.waddell@phac-aspc.gc.ca

# Important Dates:

Evidence published up to January 28, 2016

Protocol version 1, initiated January 27, 2016

Contents

[Title: 1](#_Toc447539922)

[Authors: 1](#_Toc447539923)

[Important Dates: 1](#_Toc447539924)

[Objectives of the ScR 3](#_Toc447539925)

[Study Question 3](#_Toc447539926)

[Study Sub- Questions 3](#_Toc447539927)

[Planned Study Outputs 3](#_Toc447539928)

[Methods 3](#_Toc447539929)

[Review Team Expertise and Responsibilities. 3](#_Toc447539930)

[Search Strategy 4](#_Toc447539931)

[Algorithms 4](#_Toc447539932)

[Databases 4](#_Toc447539933)

[• Scopus, PubMed/MEDLINE, Embase, CINAHL (Cumulative Index to Nursing & Allied Health), CAB, LILACS (South American), Agricola, COCHRANE library for any relevant trials in the trial registry 4](#_Toc447539934)

[Grey Literature Sources and Procedures 4](#_Toc447539935)

[Search Verification 4](#_Toc447539936)

[Relevance Screening (RS) 4](#_Toc447539937)

[Inclusion / Exclusion criteria 4](#_Toc447539938)

[Study Characterisation 5](#_Toc447539939)

[Review Management 5](#_Toc447539940)

[Data Analysis 5](#_Toc447539941)

[Appendix 1: Relevance Screening Tool 6](#_Toc447539942)

[Appendix 2: Data Characterisation Form 8](#_Toc447539943)

[Appendix 3: Search Strategy 31](#_Toc447539944)

# Objectives of the ScR

## Study Question

*What is the current state of the evidence on Zika virus pathogenesis, epidemiology, risk factors, diagnosis, surveillance methods, prevention and control strategies and, knowledge, societal attitudes and perceptions towards infections in humans, mosquito vectors and animal reservoirs?*

This research aligns with PHRSD priorities, which include enhancing and guiding public health decision-making and policies by providing the authoritative analyses, recommendations and scientific collaborative services (using methods such as epidemiological studies and knowledge synthesis) to address the occurrence, trend and determinants of infectious disease in Canada with expert focus on the prevention of public health risks arising from the food chain, animals and the environment (LFZ, 2013).

## Study Sub- Questions

The scoping review will aim to capture all published literature addressing the following aspects of Zika virus:

1) **ZikaV infection in humans, any host or vector?** (pathogenesis, epidemiology, diagnosis, conditions for virus transmission, surveillance for ZikaV)

2) **Studies on ZikaV** (e.g. pathogenesis, transmission and molecular mechanisms)

3) **Strategies to prevent ZikaV infections and/or control ZikaV harbouring vectors**

4) **Societal knowledge, perception and attitudes towards ZikaV.**

## Planned Study Outputs

1. Primary scoping study of the review findings.
2. A summary of findings fact sheet.
3. A repository and dataset of all relevant literature captured in this study.

# Methods

## Review Team Expertise and Responsibilities.

| **Member** | **Organization** | **Project Role*** |
| --- | --- | --- |
| Lisa Waddell | RISK - Guelph | Synthesis expertise- participate as needed |
| Judy Greig | RISK - Guelph | Synthesis expertise – participate as needed |
| Aamir Fazil | NML | Advisory |
| Dr. Victoria NG | NML | Advisory |
| Dr. Nick Ogden | NML | Advisory |
|  |  |  |
|  |  |  |

## Search Strategy

### Algorithms

### Databases

### Scopus, PubMed/MEDLINE, Embase, CINAHL (Cumulative Index to Nursing & Allied Health), CAB, LILACS (South American), Agricola, COCHRANE library for any relevant trials in the trial registry

### Grey Literature Sources and Procedures

***Possible sources of grey literature***

- WHO library (including SEARO, IMSEAR, IMEMR)
- CDC – MMWR, FastStats – Infectious Disease ( [www.cdc.gov/nchs/faststats/infectious -disease.htm](http://www.cdc.gov/nchs/faststats/infectious%20-disease.htm), ArboNET
- PHAC
- National Institutes of Health (NIH)
- Australia’s **National Notifiable Disease Surveillance System (NNDSS)**
- European Centre for Disease Prevention and Control (ECDC)
- PAHO
- Eurosurveillance
- ProMED-mail
- MedCarib
- Caribbean Public Health Agency (www.carpha.org)

### Search Verification

A snowball strategy for search verification will be used. This includes references being screened for potential relevance and if not already included in our ScR database, they will be added to the review for screening. At the point of saturation, when no new references are identified, we will stop evaluating reference lists.

- Screening reference lists of reviews (19) and risk assessments (3).

## Relevance Screening (RS)

The relevance screening level will be done on the title, abstract and keywords where available There is 1 question with additional detail requested if relevant. The questions are based upon the inclusion / exclusion criteria and can be found in the appendix.

### Inclusion / Exclusion criteria

***Potential inclusion/exclusion criteria***

1. Time frame – no time frame
2. Country – All
3. Language – English, French. All other languages will be identified and parked until resources and time is available. e.g Spanish or Portuguese
4. Document Type: All - any peer review primary articles, reviews, commentaries, PhD/MSc Theses, reports.

## Study Characterisation

The characterisation level of this scoping study is to first confirm relevance of a publication and second to extract all important information from a citation to understand the value and characteristics of the Zika virus study. This will include; study design, evaluation of bias, cost, timeliness, surveillance methodologies and results, reported risk factors for susceptibility to infection or environmental risk factors contributing to infection, diagnostic tests evaluations, potential prevention and control measures and their effectiveness, attitudes and perceptions towards Zika V.

## Review Management

The search strategy will be compiled and deduplicated in a RefWorks database. This database will then be exported to DistillerSR, a web-based systematic review software designed to manage all stages of conducting scoping reviews and systematic reviews. All stages of the scoping study from relevance screening to data extraction will be conducted within this software. The final dataset will be exported into MS Excel, cleaned and tabulated for use in the publication and reports.

## Data Analysis

This will likely be a descriptive tabulation of all pertinent information regarding ZikaV; including risk factors, surveillance, diagnostics, prevention and control, and perceptions and attitudes. Findings and recommendations, methods incorporated and their usefulness, and study limitations will also be captured. All knowledge gathered will be summarised and discussed.

## Appendix 1: Relevance Screening Tool

*What is the current state of the evidence on Zika virus pathogenesis, epidemiology, risk factors, diagnosis, surveillance methods, prevention and control strategies and, knowledge, societal attitudes and perceptions towards infections in humans, mosquito vectors and animal reservoirs?*

**Relevance Screening Tool for Abstracts:**

| **Question** | **Options** | **Definitions/additional notes** |
| --- | --- | --- |
| 1. Capture all studies on ZikaV.  1) **ZikaV infection in humans, any host or vector?** (pathogenesis, epidemiology, diagnosis, conditions for virus transmission, surveillance for ZikaV)  OR  2) **Studies on ZikaV** (e.g. pathogenesis, transmission and molecular mechanisms)  OR  3) **Strategies to prevent ZikaV infections and/or control ZikaV harbouring vectors**  OR  4) **Societal knowledge, perception and attitudes towards ZikaV.** |  Yes – primary research on ZikaV   No – Model on ZikaV   No – Review on ZikaV   No – Official alert/news/guidlelines on ZikaV (WHO, CDC, eurosurv)   No – Newspaper/ magazine article on ZikaV   No, not relevant (excluded, submit form)  If yes, select the type of research   - Pathogenesis of ZikaV infection= diagnosis, clinical characteristics and complications   - Humans   - Hosts - Vectors - Evaluation of diagnostic tests for ZikaV in humans, hosts or vectors - Epidemiology of Zika virus in humans hosts or vectors.   - Prevalence / incidence   - Outbreaks / sporadic cases - Risk factors for ZikaV infections in humans or hosts   - Risk factors for the ZikaV occurrence in vectors   - Conditions for ZikaV transmission between vector and host/human. - Surveillance to determine the extent of ZikaV infections in humans hosts or vectors - Zika Virus study (pathogenesis, transmission characteristics and/or molecular characterisation) - Vector adaptation/suitability for transmission of ZikaV. - Efficacy of mitigation strategies to prevent and/or control ZikaV infections in humans, hosts or vectors - Societal knowledge, attitudes and/or risk perceptions towards ZikaV and potential prevention and control strategies - Economic burden of ZikaV infections and/or cost-benefit of potential prevention and control strategies - Other ZikaV topic specify: ____ | **ZikaV** is a Flavivirus mainly transmitted to humans through bites from Aedes mosquito vectors that harbor ZikaV. **Aedes mosquitoes**, *Aedes aegypti* and *Ae. Albopictus* have been the main vector species to date.  Some human to human transmission has been documented (semen, blood transfusion, mother to fetus) and the ZikaV has been isolated in saliva and urine during viremic period.  The virus has been isolated from:  *Ae. Africanus*  *Ae. Apicoargenteus*  *Ae. Luteocephalus*  *Ae. Aegypti*  *Ae. Vitattus*  *Ae. Furcifer*  **Reservoirs** are thought to be African primates. Rodents have been shown to have a serological response ZikaV in Polynesian islands. Experimental infection of mice and monkeys has occurred.  ***Pathogenesis*** refers to the biological processes/mechanisms/pathways that lead to ZikaV disease  ***Diagnostic tests*** for detecting the presence of ZikaV in humans, other non-human hosts and vectors  ***Risk factors*** are environmental, behavioural, or biologic factors usually in longitudinal, cross-sectional, cohort or case control studies where exposures and outcomes are studied. A risk factor indicates an association with an increase or decrease in disease in the population with the risk factor compared to that without.  ***Surveillance*** is the ongoing and systematic collection, analysis, and interpretation of outcome-specific data for use in the planning, implementation, and evaluation of public health practice. Include studies evaluating surveillance methods/programs.  Examples :   - Surveillance of human cases - Sylvatic host surveillance (Sylvatic cycling is when pathogen transmission occurs between animal i.e., sylvatic hosts and vectors) - Mosquito/vector surveillance   ***Mitigation strategies = interventions***   - Studies looking at intervention efficacy include control or challenge trials and quasi experiments (before and after). - Program evaluations can fall in here. - Risk factors looking at presence/absence of an intervention should also be checked here. - Examples include (but are not limited to) land management, vector management and control, personal protection, and public education campaigns   ***Risk perceptions*** *are* the subjective judgements that people make about the characteristics and severity of a risk. Do individuals feel they are at risk? Do they have knowledge that they can implement to decrease their risk? What are their feelings concerning using sprays or treating mosquitoes to decrease the risk of disease transmission?  ***Economic burden*** will include an actual dollar amount or discussion of implied cost associated with mitigation strategies.  ***Cost benefit analysis*** is a systematic process for calculating and comparing benefits and costs of a project, decision or government policy. |

## Appendix 2: Data Characterisation Form

**Broad topic:**

*What is the current state of the evidence on Zika virus pathogenesis, epidemiology, risk factors, diagnosis, surveillance methods, prevention and control strategies and, knowledge, societal attitudes and perceptions towards infections in humans, mosquito vectors and animal reservoirs?*

**Article-level data characterisation and utility form (applicable to all relevance-confirmed articles)**

**Note:** Remember to only extract information in that applicable question – **not all questions apply.** Be very specific about the data you extract AND only **extract primary information** (information collected by the author in the course of the experiment.)

| **Question** | **Options** | **Definitions/Additional notes** |
| --- | --- | --- |
| 1. What language is the article published in? | - English - French - Spanish - Portuguese - Other, please specify: ____ (Exclude, please submit) | Due to time and $, we will proceed with Spanish and Portuguese later. |
| **If exclusion criteria were selected above, submit the form before proceeding** | | |
| 2. What type of document is this article? | - Primary research or model in peer-reviewed journal - Thesis - Grey literature with primary data (government or research reports) - Conference proceedings - Literature review (Exclude, please submit) - Systematic review/meta-analyses (Exclude, please submit) - Grey literature; may report previously reported research (newspaper or magazine articles; exclude, please submit) | **Primary research:** original research/investigation/study carried out by the researcher (incl. surveys, interviews, outbreak reports, observations, etc.)  **Thesis:** a long paper/essay or dissertation involving personal research (usually written for a university degree)  **Conference proceeding abstract/short paper:** A collection of published academic papers  **Literature review:** Examination of published literature  **Systematic review/meta-analyses:** Analysis and interpretation of primary research  **Grey literature:** Research that is unpublished or published in a non-commercial form |
| **If exclusion criteria were selected above, submit the form before proceeding** | | |
| 4. On what continent was the study conducted?  Specify the country(ies) | - North America - Europe - Australasia - Central America/South America/Caribbean - Asia - Africa - Other   Txt _______ | **North America:** Includes Canada, USA and Mexico  **Europe:** includes, Belarus, Latvia, Ukraine, Estonia, Cyprus & west (incl. Iceland and Greenland)  **Australasia:** limited to Australia, New Guinea, New Zealand, New Caledonia, and neighbouring islands, including the Indonesian islands from Lombok and Sulawesi eastward  **Central America/South America/ Caribbean:** Includes Caribbean, and all of south and central America.  **Asia:** Russia, Turkey, middle eastern countries and east  **Please specify country in the text box with full name (exception: USA)** |
| 5. When was the article published? | - Pre 1960 - 1960-1970 - 1971-1980 - 1981-1990 - 1991-2000 - 2001-2010 - 2011-Present |  |
| 6. When were samples collected or the study conducted? (This will allow aggregation of results according to timeframe) | - Text box |  |
| 7. What do the results of the paper focus on?  (Check all that apply; When answering this question, only check off the topics for which there are study outcomes and do not check if a category was just “mentioned” in the paper.) | - **Pathogenesis** of ZikaV infection= diagnosis, clinical characteristics and complications   - Treatment of ZikaV infection - Evaluation of **diagnostic tests** for ZikaV in humans, hosts or vectors - **Epidemiology** of ZikaV related to the host, vector and/or ZikaV - Conditions for ZikaV **transmission** between vector and host/human (temperature, humidity etc.). - **Surveillance** to determine the extent of ZikaV infections in humans, hosts or vectors - Zika **Virus study** (pathogenesis, transmission characteristics and/or molecular characterisation) - **Vector adaptation/**   **suitability** for transmission of ZikaV.   - Efficacy of **mitigation** strategies to prevent and/or control ZikaV infections in humans, hosts or vectors - **Societal knowledge, attitudes and/or risk perceptions** towards ZikaV and potential mitigation strategies - **Economic burden or cost-benefit** analysis of ZikaV infection and/or mitigation strategies - Other ZikaV topic (Use if absolutely necessary) | ***Pathogenesis:*** biological processes/mechanisms/  pathways that lead to ZikaV disease in human, vector or host. This includes the following:   - Infection mechanisms (at cellular level, stages of infection) including ZikaV entry/exit or inhibitors of ZikaV entry/exit - Immune response (Proteins/genes/receptors involved; in host and vector) - Pathology of disease (chronic or acute symptoms and organs affected) - Animal models studying pathogenesis - Signs and symptoms: Check if clinical signs and symptoms of ZikaV infection in humans and/or data on how to diagnose ZikaV are described in this paper   ***Diagnostic tests*** refer to tests detecting the presence of ZikaV in humans, non-human hosts or vectors.  ***Risk factors*** are environmental, behavioural, or biologic factors usually in longitudinal, cross-sectional, cohort or case control studies where exposures and outcomes are studied. A risk factor indicates an association with an increase or decrease in disease in the population with the risk factor compared to that without.  ***Epidemiology:*** Please include articles describing outbreak and sporadic cases, incidence/  prevalence for ZikaV, and/or risk factors for developing ZikaV infection or risk factors/conditions (environmental and climatic mostly) for ZikaV survival in vectors.  ***Surveillance*** is the ongoing and systematic collection, analysis, and interpretation of outcome-specific data for use in the planning, implementation, and evaluation of public health practice. Include studies evaluating surveillance methods/programs.  Examples :   - Surveillance of human cases - Sylvatic host surveillance (Sylvatic cycling is when pathogen transmission occurs between animal i.e., sylvatic hosts and vectors) - Mosquito/vector surveillance   ***ZikaV Virus studies*** include:   - Molecular characterization of ZikaV (e.g. mutations, phylogenetic analysis) - ZikaV pathogenic attributes (Describes how viruses cause disease e.g. virulence factors, viral entry/exit/cycle (includes latency period), viral replication) - ZikaV transmission (passing of virus from an infected host to another vector/host; e.g. mosquito transmission, mother to child and/or through blood transfusion) and adaptability (ability to adapt to new host/environment or become resistant to drug) - Check sylvatic-arbovirus dynamics if results discuss sylvatic cycle (In this cycle, the virus cycles between vector/arthopod and non-human hosts)   ***Vector adaptation and suitability studies for ZikaV transmission*** include:   - Insecticide resistance - Vector mortality - Characteristics of competent vector (genes, adaptations, etc...; ability to transmit disease) - Range and density of ZikaV vector and/or environmental/climatic conditions to sustain vector population - Vector activity (biting rate, Fecundity/fertility rate, reproductive rate etc.) - Extrinsic incubation period (Interval between the uptake of ZikaV by vector and vector’s ability to transmit ZikaV to other susceptible hosts) - Transmission/rate of infectivity (ie: how many people could be exposed by one infected mosquito and how many mosquitos are likely to become infected by one viraemic human)   ***Mitigation strategies = interventions***   - Studies looking at intervention efficacy include control or challenge trials and quasi experiments (before and after). - Program evaluations can fall in here. - Risk factors looking at presence/absence of an intervention should also be checked here. - Examples include (but are not limited to) land management, vector management and control, personal protection, and public education campaigns   ***Risk perceptions*** are the subjective judgements that people make about the characteristics and severity of a risk. Do individuals feel they are at risk? Do they have knowledge that they can implement to decrease their risk? What are their feelings concerning using sprays or treating mosquitoes to decrease the risk of disease transmission?  ***Economic burden*** will include an actual dollar amount or discussion of implied cost associated with mitigation strategies.  ***Cost benefit analysis*** is a systematic process for calculating and comparing benefits and costs of a project, decision or government policy.  ***Predictive models*** are mathematical or statistical models used to forecast outcomes, spread of ZikaV and/or trends. Examples include (but are not limited to) using climate to predict outbreaks and/or models predicting high-risk populations. In the provided text box, please describe model in one line. If possible, copy and paste text from the abstract/objectives section. |
| 8. What is the study design?  *(Check all that apply)* | - Observational study   - Cross-sectional   - Cohort   - Case-control   - Prevalence survey   - Surveillance or monitoring program   - Case study or case-series   - Outbreak investigation   - Sporadic cases investigation   - Longitudinal study   - Evaluation of Diagnostic Tests   - Other, specify: _____ - Experimental study - Controlled trial - Challenge trial - Quasi experiment - Other, specify:_____ - Qualitative study, specify:________ - Mixed methods - Economic model - Disease transmission model - Risk assessment - Vector mapping model - Other, please specify: ___ - N/A (review or commentary; Stop reviewing and submit form) | **Observational study**: Assignment of subjects into a treated group versus a control group is outside the control of the investigator.   - **Cross-sectional:** Examines the relationship of a risk factor and outcome (disease) at a point in time on representative samples of the target population. - **Cohort study**: is a study in which individuals with differing exposures to a suspected risk factor are observed through time for occurrence of an outcome - **Case-control study**: compares exposure to the risk factor in subjects who have an outcome (the 'cases') with subjects who do not have the outcome, but are otherwise similar (the 'controls') and drawn from the same sampling frame. - **Prevalence survey:** Measurement of an outcome at a point in time but doesn’t measure or investigate potential predictors – include here routine monitoring or surveillance data collection - **Case or case-series:** a descriptive study of a single individual (case report) or small group (case series). - **Outbreak investigation:** Studies an outbreak retrospectively or while it is occurring. An outbreak is a sudden increase in the occurrence of CHIKV illness in a given area with linked cases - **Sporadic cases investigation:** Studies cases of CHIKV infection that are not linked in space and/or time. - **Longitudinal study:** A research method in which data is gathered for the same subjects over a period of time.   **Evaluation of Diagnostic Tests:** One or more diagnostic test is evaluated for sensitivity, specificity against a gold standard, clinical symptoms or another test.  **Experimental study:** Each subject is assigned to a treated group or a control group before the start of the treatment   - **Control trial:** an experimental study in which people are allocated to intervention groups and evaluated for outcomes. - **Challenge trial:** An experiment where subjects are artificially challenged or exposed to the disease agent and then allocated to the intervention groups for evaluation of the outcome. - **Quasi-experiment:** An experiment in which subjects are not randomly assigned to groups. Often this is the method of choice in field trials where the samples of the outcome are taken from the same individuals before and after the experiment/intervention   **Qualitative study:** Aimed at understanding social phenomena, exploring issues, and answering questions of “why” and “how.” Please specify the design/methodology that is identified by the author, and if none is identified explicitly then indicate “not specified”  **Mixed methods:** Tackles a research question using different research methodologies.  **Economic models** use mathematical equations to describe how costs are affected by different inputs. The structure of the equations reflects the model builder’s attempt to describe reality.  **Disease transmission models** are mathematical models used to link the biological process of transmission and the emergent dynamics of infection at the population level.  **Risk assessment** is the determination of quantitative or qualitative value of risk related to a situation and a recognized threat (hazard). Quantitative risk assessment requires calculation of the magnitude of the potential loss and the probability that the loss will occur.  **Vector mapping** is collection of data on spread/range of vector population (may use GIS) |
| **If exclusion criteria were selected above, submit the form before proceeding** | | |
| 9. What vector or host species were studied in this article? (Including the number and type of samples for humans)  *(Check all that apply)* | ** Humans**  **Samples taken to test for ZikaV**:   - - Blood   - Semen   - Urine   - Saliva   - Other, please specify_____   - Questionnaire/focus group   **Characterize the human population ( or case report) sampled for ZikaV**   General population   Paediatric   - Adults - Elderly   **Immunocompromised**   - Drug induced - Physiological - Pregnant - Other, specify: ____   **Co-infection**   - Dengue and/or CHIKV - Other, specify: ___   **Co-morbidity**   - Diabetes - Cardiovascular - Other, specify: ___   ** Other non-human host (reservoir) species _____**   - species **investigated** for sylvatic cycle transmission    species **identified** to be part of the sylvatic cycle; please specify species: ___  ** Mosquitoes**  *Aedes aegypti*  *Aedes Africanus*  *Aedes albopictus*  *Aedes apicoargenteus*  *Aedes dalzieli*  * Aedes tunestus*  *Aedes furcifer*  *Aedes hensilii*  *Aedes luteocephalus*  * Aedes polynesiensis*  *Aedes Vitattus*  *Culex* species, specify: ____  Other, specify: ___  ** Non-mosquito arthropod vectors please specify species______**    ** Animal model**   Mice   Non-human primates, please specify____   Other, please specify: _____  ** Virus only studies using cell-cultures, in-vitro models** | Population sampled for Zika refers to age range of the population studied. If it is a case report, specify age in epidemiology section.   - **General population (**0-infinity). No focus on a specific age group - **Specific population** selected when authors differentiate between age groups. For the purpose of this review: - **Pediatric** < 16 - **Adults**: 16 to 65 - **Elderly** >65   **Immunocompromised** Examples are persons with weakened immune systems include those with AIDS; cancer and transplant patients who are taking certain immunosuppressive drugs; and those with inherited diseases that affect the immune system   - **Drug induced**: Includes steroid treatment - **Physiological** is due to gene defect/spleen removed. It can be congenital or due to infections such as HIV.   In the **sylvatic cycle**, the virus cycles between vector/arthopod and non-human hosts  **Non-mosquito arthropods** could include ticks, fleas and other insects. |
| 10. What is the molecular characterization of ZikaV as described by the authors?  *(Check all that apply)* | **Genotypes**   Asian lineage   African lineage, not specified   West African lineage (Senegal/Nigerian)   East/Central/South African lineage (Uganda)   Other, please specify: ____   Not reported   Not applicable, no molecular characterisation or no Zika.  **Are any mutations reported?** (specify as reported by author)  ______________________________ | **Not reported:** Authors did test molecular characterization of ZikaV but didn’t report it.  **Not applicable:** Authors did no testing to determine molecular characterization of ZikaV.  **Not reported:** Mutation not reported. Only looked at it at the macro level |
| If pathogenesis - signs/symptoms is selected | | |
| 11. Does this study report on travel-related cases? |  Yes, Symptoms develop while person is in visiting country, specify visiting country:_____   Yes, Symptoms develop after person is back from country where ZikaV was acquired, specify person’s country of origin:_____  No |  |
| 12. Reported signs and symptoms of ZikaV infection. (Format: +ve/N  /time units and comments)  *(Check all that apply)* |  Fever, specify: ________   Joint pain, specify : ________   Rash, specify : ________   Conjunctivitis (red eyes): ____   Muscle pain, specify: ________   Headache, specify: ________   Other, please specify: ________ | When specifying, state the number of cases with symptom(s), number of total cases, and duration of time with units and further comments. Order should be (#/#/time + units/comments)  If duration of illness in many patients is provided, state the range and mention that “individual patient data is available”. |
| 13. How was ZikaV diagnosed in humans included in this study?  *(check all that apply)* |  Based on clinical symptoms  Virus isolation  RT-PCR (reverse transcription PCR)   Serology   - IgG - IgM - Plaque reduction neutralization tests - Other, specify; ____    Molecular characterization   Other, specify: ______ | There are no commercially available diagnostic tests for ZikaV. |
| 14. Are sequalae reported following ZikaV infection?  (Format: +ve/N/time units and comments)  *(Check all that apply)*  For the sequelae reported, what are the characteristics of the condition and risk factors for getting the condition as reported by the author |  Birth defects:   - Microcephaly - Other poor pregnancy outcomes, specify: _____    Guillain-Barré syndrome (GBS), please specify ___________   Other, please specify: ______   Characteristics: please specify ___________   Risk Factors for getting the above mentioned sequalae, please specify: ______ | When specifying, state the number of cases with symptom(s), number of total cases, duration of time with units and further comments. Order should be (#/#/time + units/  comments)  **Microcephaly** is a condition in which a baby’s head is smaller than expected when compared to babies of the same sex and age.  **Guillain-Barré syndrome** is a rare disorder where a person’s own immune system damages the nerve cells, causing muscle weakness and sometimes, paralysis. |
| Treatment questions |  |  |
| 15. What treatment options were used to treat ZikaV infections? |  Plant-based inhibitors, specify: _____   Non-steriodal anti-inflammatory drugs, specify: ______   Corticosteroids, specify: _________   Analgesics /anti-pyretic, specify: _________   Anti-viral drugs, specify: _________  Physical therapy or acupuncture, specify: _________  Traditional medicine, specify: ____   Other, specify: __________ | Specify name of drug  **Analgesics** is a group of drugs that is used to relieve pain. Examples include Tylenol, Parcetamol and Advil.  **Anti-pyretic drugs** are used to prevent/reduce fever.  **Anti-viral drugs** is a group of drugs used to treat viral infections by inhibiting development of virus.  **Traditional medicine** includes Chinese medicine, homeopathy and ayurvedic medicine. |
| 16. What treatment options were evaluated for efficacy? |  Plant-based inhibitors, specify: _____   Non-steriodal anti-inflammatory drugs, specify: ____________   Corticosteroids, specify: _________   Analgesics /anti-pyretics, specify: _________   Anti-viral drugs, specify: _________  Physical therapy or acupuncture, specify: _________  Traditional medicine, specify: ____   Other, specify: __________ | Specify name of drug, and whether it was effective or non-effective as evaluated by author.  This question will be directed towards experimental studies. |
| If accuracy of diagnostic tests is selected | | |
| 17. What tests were examined for their accuracy in the diagnosis of human cases and/or detection of ZikaV in non-human hosts?  *(Please check all that apply)* | - Clinical diagnosis (by signs and symptoms) - Clinical diagnosis is a referent group for any test in the paper. - Virus culture and identification - Serological Tests   - IgG   - IgM   - Plaque reduction neutralization tests   - Other serological test, specify: ___ - Molecular Tests   - RT-PCR (reverse transcription PCR)   - Other molecular tests, specify: __ - Other, specify:___ | **Plaque reduction neutralization tests** measure neutralizing antibodies for ZikaV  **RT-PCR:** Used to qualitatively detect gene expression through creation of complementary DNA transcripts from RNA |
| 18. Is information about sensitivity, specificity and/or raw data provided? | - Yes, specify the test(s): _____   What data is available?   - Specificity is provided - Sensitivity is provided - Raw data is provided (for 2 by 2 table) - Detection limits of test, - When should this test be used? (e.g. X days after symptoms appear) Specify: ____   **Is this a commercial test?**   - Yes - No, it an inhouse or experimental test - No, insufficient data provided | **Sensitivity** (also called the **true positive rate**) measures the proportion of positives that are correctly identified as such.  **Specificity** (also called the **true negative rate**) measures the proportion of negatives that are correctly identified as such.Eg., if 100 people known to have a disease were tested and 43 tested positive, the test has 43% sensitivity. If 100 people with no disease are tested and 96 return a negative result, then the test has **96%** specificity.  **Detection limits** – examples are cut off values for detecting positive or negative results for each test  If information about SN/SP/raw data/limitations of test, etc.. is provided for more than one test, select “Yes, for multiple tests” and more questions will become available. |
| 19. Additional Comments about diagnostic tests in this paper | Textbox |  |
| If Epidemiology is selected | | |
| 20. What is the **burden** of ZikaV in humans?  (Only answer if the data is a population sample.) |  The sample represents [ date/ region/ population] = ____________   Prevalence (if reported) = __________   Incidence (if reported) = ______   Long-term sequelae = ____   Case-fatality rate = ____   Rate of fetal death attributed to ZikaV = ____   Rate of microcephaly attributed to ZikaV = ____   Other ____ | **Sample:** describe what the sample represents – date, region, population sampled.  **Prevalence:** It is the number of cases of ZikaV in a defined population at a specific point in time. Record both numerator and denominator if provided [# of total ZikaV cases at a point in time, # of exposed individuals). If number of asymptomatic cases are reported please include in text box.  **Incidence:** It is the number of new cases of ZikaV arising within a given time period in a specified population. Record both numerator and denominator if provided [# of new ZikaV cases in a given time period, # of exposed individuals)  **Long-term sequalae:** Proportion of cases that develop chronic symptoms e.g. Guillain-Barré syndrome (GBS),  **Case-fatality rate:** Proportion of cases that die from all ZikaV cases |
| 21. Characteristics of ZikaV infection in humans:  (Only answer if primary data is available) |  Time between exposure and viraemic period, please specify in days ______   Viremic period, please specify in days: ___   Intrinsic incubation period (IIP), please specify in days: ___  Other ____ | **The intrinsic incubation period (IIP)** is the time between a human being infected and the onset of symptoms due to the infection.  **Viraemic period** is the time period in which humans are infectious with ZikaV. |
| 22. What is the burden of ZikaV in mosquito vectors? (Only answer if the data is a population sample.) |  The sample represents [ date/ region/ population] = ____________   Prevalence, specify: ___   Co-infection, specify: ____   Other measure of burden of infection, specify: ____ | **Sample:** describe what the sample represents – date, region, population sampled.  For the **prevalence,** include the species name, number captured and the number of positive ZikaV mosquitoes as [species, #positive, # captured]  For **co-infection**, specify the name of the co-infection, only capture any human illness causing virus (e.g., Dengue). |
| 23. What is the burden of ZikaV in non-human animal hosts? (Only answer if the data is a population sample.) |  The sample represents [ date/ region/ population] = ____________   Prevalence, specify: ___   Co-infection, specify: ____   Other measure of burden of infection, specify: ____ | **Sample:** describe what the sample represents – date, region, population sampled.  For the **prevalence,** include the species name, number tested and the number of positive ZikaV mosquitoes as [species, # tested,#positive]  For **co-infection**, specify the name of the co-infection |
| 24. Describe the human ZikaV **outbreak**  (Only answer if this is an outbreak report) |  Outbreak cases; total number reported:____   - Outbreak start date (yyyy/mm/dd): ____ - Outbreak finish date (yyyy/mm/dd): ____ - Number of confirmed cases:____ - Number of probable cases:____ - Number of hospitalizations: ___ - Number of fatalities: ____ - Number of stillbirths __ - Mother-to-child transmission -  Number of pregnant cases; please specify: ____ - Number of newborn cases, please specify, ___   **Describe the infection sequalae: please specify number of cases**   - Birth defects: Microcephaly ______ - Birth Defects: Other poor pregnancy outcomes, please specify and record number affected: _____    Guillain-Barré syndrome (GBS):___   Other, please specify and record number affected: ______  **For the sequelae associated with Zika Virus, what are the characteristics of the condition and risk factors for getting the condition as reported by the author**   Characteristics: please specify ___________   Risk Factors for getting the above mentioned sequalae, please specify: ______ | **Confirmed cases** include all ZikaV cases that are laboratory confirmed.  **Probable cases** are cases that are clinically diagnosed without laboratory confirmation |
| 25. Describe the ZikaV sporadic cases.  (Only answer if this is a sporadic case report) |  Sporadic cases; total number reported:____   - Number of confirmed cases:____ - Number of probable cases:____    Mother-to-child transmission   Number of pregnant cases; please specify, ____   Number of newborn cases, please specify, _______ | **Sporadic cases** = When you see cases here and there. There is nothing linking one case to another. |
| 26. Does this study report on travel-related cases? | Yes   Symptoms develop while person is in visiting country, specify visiting country:_____   Symptoms develop after person is back from country where ZikaV was acquired, specify person’s country of origin:_____  No |  |
| 27. What risk factors were investigated for humans developing **ZikaV infection** (ie: sampled and tested, not just mentioned)  (Only applies to epidemiology studies: surveys, cross sectional, case control, cohort. Not outbreak investigations) | - Text box ______________ | Copy and paste from text or list the risk factor, direction of association and whether significant or not |
| **For the sequelae (e.g. microencaphaly) associated with Zika virus, what are the risk factors for getting the condition as reported by the author?** |  Characteristics: please specify ___________   Risk Factors for getting the above mentioned sequalae, please specify: ______ |  |
| Conditions for ZikaV transmission between vector and host/human | | |
| 29. If transmission was human to human please describe method: | - Intrauterine   - Stage of pregnancy when infection occurred _____ - Sexually transmitted - Blood transfusion - Other, please specify ______ |  |
| 30. Which bodily fluid was associated with human-to-human transmission? | - Saliva - Blood - Semen - Urine - Other, please specify ____ - Number of days ZikaV viable in these fluids ________ as reported |  |
| 31. Is the transmissibility between humans and mosquitoes described? | - Yes humans to mosquitoes - Yes mosquitoes to humans - No | **Human-to-mosquito transmissibility** is the probability of a mosquito acquiring ZikaV from an infectious human/host during a single blood meal.  **Mosquito to human/host transmissibility** is the probability of a human/host acquiring ZikaV from an infected mosquito during a single blood meal |
| 32. Is the transmissibility between non- human hosts (reservoirs) and mosquitoes described? | - Yes reservoir to mosquitoes - Yes mosquitoes to reservoir - No |  |
| Direct transmission between host and human, please describe: | text | e.g. monkey bite |
| If Surveillance is selected | | |
| 33. What is the goal of the surveillance system/program? | Text box | Please copy and paste the goal of the surveillance system. For example, to identify the number of mother-to-child ZikaV transmissions. |
| 34. Describe region under surveillance | Textbox | Describe area (i.e. urban/rural) and comment on scale of it (i.e. size of area sampled) |
| 35. When was the surveillance program initiated/finished? | Start date: (yyyy/mm/dd)  End date: (yyyy/mm/dd) | State NA if not provided. If it’s still on-going, write “on-going” in the end date section |
| 36. What surveillance methods are described?  *(Check all that apply)* |  Active:   Targeted sampling protocol used, specify: ____   Monitoring program   Targeted sampling protocol used, specify: ____   Passive   Physician reporting___   Laboratory-based ___   Event-based ___   Other, specify: _____ | **Sampling protocol:** Describe the sampling strategy used. Ex. 10 shipping containers checked, 200 km^2^ area searched  **Active surveillance**, in contrast to passive surveillance, requires that public health staff take direct action to collect disease information. For example, they may contact physicians, hospitals, laboratories, or other health entities to actively search for disease cases. Active surveillance may also occur through direct review of clinical or hospital charts, laboratory records, or emergency room patient logs. Active surveillance provides the most complete picture of disease incidence, i.e., cases are found in a timely manner, a greater number of cases are found, and more thorough information is obtained compared to passive surveillance methods. Active surveillance is an on-going activity and contains thresholds.  **Monitoring program:** Systematic purposeful program without active action plan. Simply counts numbers.  With **passive surveillance**, a member of the reporting community initiates a disease report that is communicated to a health department. For example, a physician may telephone a health department to discuss a case immediately upon seeing a patient with a suspected or confirmed case of a disease or an infection control practitioner may contact a health department upon receipt of positive laboratory results for a more common disease.  **Laboratory surveillance** differs from population-wide surveillance in that it can only monitor patients who are already receiving medical treatment and having lab tests done - does not identify patients who have never been tested.  **Event based surveillance** refers to the aggregation of data resulting from the monitoring of internet sourses such as ProMed and GPHIN |
| 37. Did the author evaluate the surveillance program? |  Yes, specify: ____   No | **Specify** results of evaluation briefly |
| If ZikaV study is selected: | | |
| 38. Describe the Zika Virus pathogenesis study | - Please describe what pathogenic activity of the virus is investigated_____ | Copy and paste the text referring to the target of the study. |
| 39. Does the research describe transmission characteristics of the virus? | - Viral entry into cells - Viral exit from cells - Virulence factors - Other, please describe _____ |  |
| 40. Does the research describe the molecular characterization of the ZikaV? | - virus sequenced - evolution of the virus discussed - phylogeny reported with a dentogram - Other, please describe _____ |  |
| If vector adaptation/suitability for transmission of ZikaV selected: | | |
| 41. What method(s) were used to trap and/or observe the vectors? | Traps   - Ovitraps - Ovitrap with odour-based attractant, please specify: __ - Ovitrap with other attractant, please specify: ___ - Ovitrap without bait - Other trap, specify: ___ - Trap with odour-based attractant, please specify: ____ - Trap with other attractant, please specify: ___ - Trap without bait   Baits only   - Odour-based attractant, please specify: ___ - Other attractant, please specify: ___    Other: _____ |  |
| 42. Did the study examine vector competence, biology and/or transmission?  (Only answer this question if there is some data/ range / measurement for the outcome specified.) |  Yes, measured in what species, please specify: ______  Yes, competence of ZikaV vectors   - Adult longevity/lifespan in days, please specify:______ - Temperature, specify: ___ - Humidity, specify: ___ - Precipitation, specify: ___ - Mosquito density per human, please specify: _____ - Temperature, specify: ____ - Humidity, specify: ____ - Precipitation, specify: ____ - Yes, range of mosquito habitats, specify: ______ - Temperature, specify: ___ - Humidity, specify: ___ - Precipitation, specify: ___ - Egg diapause, please specify: _____ - Temperature, specify: ___ - Humidity, specify: ___ - Precipitation, specify: ___ - Female fecundity rate, please specify:____ - Temperature, specify: ___ - Humidity, specify: ___ - Precipitation, specify: ____ - Egg hatching rate, please specify: ____ - Temperature, specify: __ - Humidity, specify: ____ - Precipitation, specify: ____ - Extrinsic incubation period, please specify in days: _____ - Temperature, specify: ____ - Humidity, specify: ____ - Precipitation, specify: ___ - Proportion of mosquitoes surviving the EIP - Temperature, specify: ____ - Humidity, specify: ____ - Precipitation, specify: ____ - Time for emergence to next stage - Larvae, specify:___ - Temperature, specify: ___ - Humidity, specify: ___ - Precipitation, specify: ___ - Pupae, specify: ___ - Temperature, specify: ___ - Humidity, specify: ___ - Precipitation, specify: ___ - Adults, specify: ____ - Temperature, specify: __ - Humidity, specify: ___Precipitation, specify: ___    Vector behaviour   Yes, feeding behaviour, please specify ____   Host biting   - Time of biting activity (night or day): ___ - Biting Rate: _____ | **Competence of ZikaV vectors:** Characteristics of vector that allow it to transmit ZikaV. This includes mosquito lifespan, female fecundity rate and extrinsic incubation period. If provided, specify the temperature, humidity and precipitation range for the different competence factors.  Specify **lifespan** in days. E.g. 4 days  **Mosquito density per human** is the number of mosquitoes per human in the population being modelled.  Specify density. E.g. 100 mosquito/human  Specify **range**. E.g. 10 km  **Egg diapause** is a physiological state of dormancy in which development is delayed. It is a mechanism used by eggs to survive unfavourable environmental conditions. Specify duration of diapause. E.g. 1 month  Specify female **fecundity rate** (number of eggs laid per female mosquito)  Specify **hatching rate:** The time needed for eggs to hatch  **Extrinsic incubation period** is the time required for a pathogen to spread from the mosquito’s gut where the virus is first present to the salivary glands where the virus can be subsequently transmitted.  **Feeding behaviour** can include information on where the mosquito prefers to bite (e.g. ankle), if the species prefers humans to animals or if they have multiple blood feeds per feeding session.  **Time of biting activity** is whether day or nocturnal biting pattern  **Biting Rate** is the number of bites on a human, per mosquito, within a given time period. |
| 43. What **mosquito exposure/abundance** risk factors were investigated (ie: sampled and tested, not just mentioned)?  (Only applies to epidemiology studies: surveys, cross sectional, case control, cohort. Not outbreak investigations)  *(Please check all that apply)* | - Text box | Copy and paste from text or list the risk factor, direction of association and whether significant or not |
| If mitigation strategies is selected | | |
| 44. What prevention/  control strategies were investigated ?  *(Please check all that apply)* | - Risk posed by blood transfusion:   - Refusal of donors if reported recent travel to ZikaV outbreak region   - Treatment of blood to inactivate the ZikaV   - Test and discard blood   - Other please specify _____    Human behavioural protective measures:   Wearing long pants and/or lightly-coloured clothing   Tucking pants into socks   Using repellents; please specify ___   Wearing clothing treated with permethrin insecticide   Using mosquito/bed nets  Having window/door screens   Emptying standing water from containers such as flowerpots or buckets and cleaning them   Removing/destroying vector habitats (e.g. containers/tires)  Other behavioural measure, _____   Chemical control measures   Use of **Insecticides**   - **Ovicide, please specify: ______** - **Larvicide, please specify: ______** - **Pupicide, please specify: ______** - **Adulticide, please specify: ________** - **Lethal ovitraps**    Other chemical control measure, specify:   Biologic control of mosquitoes  Sterile insect technique (SIT)  Incompatible insect technique (IIT)/Cytoplasmic incompatability   - Infection with an endosymbiotic bacterium - *Wolbachia,*  specify type: ___ - Other bacterium, specify: ___    Release of Insects with Dominant Lethal [RIDL] mosquitoes  Use of larvivorous fish/copepods species; please specify----------  Other biological control of mosquito, specify: _____   Public education to decrease risk of ZikaV disease: please specify___   Other, please specify ______ | **Specify** name and description in text box in the order, [name, description]  In the text box, include [commercial name, description]  **Ovicide** includes insecticides targeted at eggs. Dessicants would be placed here.  **Larvae** are hatched.  **Pupae** don’t have wings but can eat.  **Adults** are fully developed (have a head with two large compound eyes, a thorax, a pair of scaled wings, and six jointed legs). Adult mosquitoes mate within the first few days after emerging.  **Lethal ovitraps** or oviposition traps incorporate an insecticide on the oviposition substrate. These traps collect the eggs laid by mosquitoes.  Different types of **larvivorous fish** feed on immature stages of mosquitoes (e.g. *Gambusia affinis, Poecilia reticulate*), thereby controlling vector population. **Copepods** are small crustaceans found in both saltwater and freshwater environments. Predatory copepods consume mosquitoes.  **Sterile insect technique** involves releasing sterile mosquitoes into the environment to mate with native mosquito vectors. Insects are usually sterilized with irradiation.  **Incompatabile insect technique/ Cytoplasmic incompatability** results in egg and sperm being unable to form viable offspring. This is caused by changes in gametes due to intracellular parasites.  **Wolbachia** is a bacterium inducing male-killing, feminization, and cytoplasmic incompatibility  RIDL uses modern molecular biology techniques to insert lethal genes into insects. Sterile transgenic insects can then be used to control mosquitoes. |
| 45. Did the authors describe the impact of the mitigation strategy? | - Successes/positive impact: _____ - Limitation/negative impact: _____ | Was the prevention/control method successful?  Insecticide resistance is a limitation. Discuss insecticide efficiency and resistance information here. |
| If social impact papers is selected | | |
| 46. Did the paper investigate knowledge and attitudes and/or risk perceptions towards ZikaV disease and potential prevention and control strategies?  *(please check all that apply)* | □ Yes, concerns about toxic or environmental effects of control measures (e.g. DEET)  □ Yes, perceptions about the severity of ZikaV disease or vulnerabilities  □ Yes, perceived efficacy of protective measures  □ Yes, knowledge on behavioural mitigation practices  □ Yes, knowledge on ZikaV disease  □ Yes, knowledge on ZikaV harbouring vectors  □ Yes, public attitudes towards paying for protection from ZikaV disease (willingness to pay)  □ Yes, other: __________ |  |
| 47. What specific populations were investigated for contextual information?  *(please check all that apply)* | □ General public  □ Physicians  □ Other medical or public health professionals, please specify:________  □ Government personnel, please specify ____  □ NGO personnel, please specify ___  □ Other, please specify _____ | What populations did the researchers speak to? Gather information from? |
| 48. How were the contextual data collected?  *(please check all that apply)* | □ Quantitative questionnaire or survey:  □ In-person, specify details _____  □ Phone, specify details _____  □ Postal questionnaire, specify details _____  □ Web-based questionnaire, specify details _____  □ Qualitative interviews:  □ In-person, specify details _____  □ Phone, specify details _____  □ Postal questionnaire, specify details _____  □ Web-based questionnaire, specify details _____  □ Focus groups  □ Analysis of documents, specify details ____  □ Other, please specify ______  □ Not specified |  |
| 49. Was the contextual data collection informed by one or more theories of human behaviour? |  Yes, Health Belief Model   Yes, Stages of Change Theory   Yes, Theory of Planned Behaviour   Other, specify _________   No | **Health Belief Model**: Authors report that the intervention changed the participants' self-efficacy and/or perceived barriers/threats to changing their behaviour  **Stages of Change Theory**, Authors report how the intervention changed the participants’ reported/observed “stages of change” classification.  **Theory of Planned Behaviour**: Did the study identify a preconceived attitude, subjective norm, and/or perceived behaviour control among participants that was addressed by the intervention, which in turn altered their behaviour? |
| If economic burden is selected | | |
| 50. Does the article report on the economic burden of ZikaV disease or cost-benefit of control measures?  (Check all that apply) |  Yes, economic burden   Descriptive, specify: ____ _______   specific cost estimates/numbers, please copy details _____   Yes, cost-benefit of control measures   Descriptive: please specify type _______   specific cost estimates/numbers: please copy details _________   Other economic measure ____   No |  |
| If other ZikaV topics are selected: | | |
| 51. What other ZikaV topic was discussed in the research article? | - Text box |  |
| Final General Questions | | |
| Is there **sufficient data** in this paper to proceed to quality assessment and further data extraction? | - Yes - No | Quick QA on whether study is worth progressing to QA/DE levels and more indepth analysis. |
| 52. Additional comments | - Textbox |  |

## Appendix 3: Search Strategy

Date of Search: January 27, 2016

| Database/ Plateform  Health Canada Library | Zika (title, keywords or abstract) | Update Feb29/16 |
| --- | --- | --- |
| Pubmed (zika a.f.) NOT (zika a.u.) | 169 | 103 |
| Scopus | 224 | 35 |
| OVID= CAB/ Agricola/EMBASE/Global health | 366 | 143 |
| Proquest | 227 | 100 |
| COCHRANE | 0 | 0 |
| LILACS- <http://pesquisa.bvsalud.org/> (tw:("zika")) | 152 | 47 |
| CINAHL | 31 | 35 |

Total citations 1169, after deduplication 525 article uploaded – January 28, 2016.

Additional 21 duplicates were detected and 1 erratum to a captured article were quarantined –

Search Verification:

The reference lists of 3 rapid risk assessments {{1902 European Centre for Disease Prevention and Control 2016, January 21; 1970 European Centre for Disease Prevention and Control 2015; 1901 European Centre for Disease Prevention and Control 2015, May 25;}} and reference lists of 16 recent and older literature reviews were examined for references not captured in our electronic search. {{247 Nhan,T.-X. 2015; 252 Yasri,S. 2015; 257 Musso,D. 2015; 258 Rodríguez-Morales,A.J. 2015; 261 Salim Mattar,V. 2015; 263 Joob,B. 2015; 274 Derraik,J.G. 2015; 358 Kuno,G. 2007; 371 Kilbourn,A.M. 2003; 374 Wolfe,N.D. 2001; 388 Pierre,V. 1994; 275 Martinez-Pulgarin,D.F. 2015; 380 Baba,S.S. 1998; 395 Buckley,A. 1988; 400 Olson,J.G. 1983; 1961 Pan American Health Organization 2015;}}. There was a lot of overlap between the references identified by examining reference lists of the above 19 publications and the search of the websites below. 34 unique citations were identified by the reference lists.

Websites of World Health Organisation (http://www.who.int/csr/disease/zika/en/), Pan American Health Organisation (http://www.paho.org/hq/index.php?option=com_content&view=article&id=11585&Itemid=41688&lang=en), Center for Disease Control and Prevention (http://www.cdc.gov/zika/index.html), Morbidity and Mortality Weekly Report (http://www.cdc.gov/mmwr/zika_reports.html), European Center for Disease Control (http://ecdc.europa.eu/en/healthtopics/zika_virus_infection/Pages/index.aspx) and ProMed-mail (<http://www.promedmail.org/>) for primary research reports, guidelines, epidemiological alerts, situation reports, surveillance bulletins and referenced publications that were not already captured. 84 additional references were added to the project, many of these were guidelines / government reports and new articles that have not been indexed in the bibliographic databases yet.

Total 149 references were identified by the search verification and grey literature search for primary information related to Zika virus.

**Update Search**

Total citations uploaded February 29, 2016= 294, removed an additional 34 duplicates= 260 to screen

March 1, 2016 added 45 additional citations from Scopus and OVID search. An additional 10 duplicates were removed.

TOTAL citations identified in update, grey literature and search verification after deduplication= 293
